# Meta-analysis of proteomics data from osteoblasts, bone, and blood: Insights into druggable targets, active factors, and potential biomarkers for bone biomaterial design

**DOI:** 10.1177/20417314241295332

**Published:** 2024-11-29

**Authors:** Johannes R Schmidt, Klaudia Adamowicz, Lis Arend, Jörg Lehmann, Markus List, Patrina SP Poh, Jan Baumbach, Stefan Kalkhof, Tanja Laske

**Affiliations:** 1 Department of Preclinical Development and Validation, Fraunhofer Institute for Cell Therapy and Immunology IZI, Leipzig, Germany; 2 Institute for Computational Systems Biology, University of Hamburg, Hamburg, Germany; 3 Data Science in Systems Biology, TUM School of Life Sciences, Technical University of Munich, Freising, Germany; 4 Fraunhofer Cluster of Excellence Immune-Mediated Diseases, Frankfurt/Main, Hannover, Leipzig, Germany; 5 Munich Data Science Institute, Technical University of Munich, Garching, Germany; 6 Berlin Institute of Health at Charité – Universitätsmedizin Berlin, Julius Wolff Institute, Berlin, Germany; 7 Computational Biomedicine Lab, Department of Mathematics and Computer Science, University of Southern Denmark, Odense, Denmark; 8 Institute for Bioanalysis, University of Applied Science Coburg, Coburg, Germany

**Keywords:** biomaterial, bone regeneration, module mining, drug repurposing, biomarker, proteomics, osteoblasts, blood plasma, extracellular vesicles, network enrichment, meta-analysis

## Abstract

Non-healing bone defects are a pressing public health concern accounting for one main cause for decreased life expectancy and quality. An aging population accompanied with increasing incidence of comorbidities, foreshadows a worsening of this socio-economic problem. Conventional treatments for non-healing bone defects prove ineffective for 5%–10% of fractures. Those challenges not only increase the patient’s burden but also complicate medical intervention, underscoring the need for more effective treatment strategies and identification of patients at risk before treatment selection. To address this, our proteomic meta-analysis aims to identify universally affected proteins and functions in the context of bone regeneration that can be utilized as novel bioactive biomaterial functionalizations, drug targets or therapeutics as well as analytical endpoints, or biomarkers in implant design and testing, respectively. We compiled 29 proteomic studies covering cellular models, extracellular vesicles, extracellular matrix, bone tissue, and liquid-biopsies to address different tissue hierarchies and species. An innovative, integrated framework consisting of data harmonization, candidate protein selection, network construction, and functional enrichment as well as drug repurposing and protein scoring metrics was developed. To make this framework widely applicable to other research questions, we have published a detailed tutorial of our meta-analysis process. We identified 51 proteins that are potentially important for bone healing. This includes well-known ECM components such as collagens, fibronectin and periostin, and proteins less explored in bone biology like YWHAE, HSPG2, CCN1, HTRA1, IGFBP7, LGALS1, TGFBI, C3, SERPINA1, and ANXA1 that might be utilized in future bone biomaterial development. Furthermore, we discovered the compounds trifluoperazine, phenethyl isothiocyanate, quercetin, and artenimol, which target key proteins such as S100A4, YWHAZ, MMP2, and TPM4 providing the option to manipulate undesired processes in bone regeneration. This may open new ways for treatment options to face the increasing socio-economic pressure of non-healing bone defects.

## Introduction

Bone fractures are becoming a more common health concern. According to a study, there were 178 million new instances of bone fractures globally in 2019, an increase of more than 33 % since 1990.^1^ Although bones possess a high intrinsic regenerative capacity the severity or complexity of the injury may exceed the bone’s natural healing abilities, necessitating additional clinical intervention for successful recovery. Additionally, age-related comorbidities or further pathologic conditions like diabetes may attenuate the self-healing capacities, as recently reviewed by Tanios et al.,^2^ making fracture healing disturbances one main cause of permanent disability, impaired quality of life and life expectancy.^3^

Already today, bone grafts/bone graft substitutes to fill and repair fractures are in clinical use to augment healing.^4^ Bone graft substitutes are often composed of natural or natural-like components (e.g., hydroxyapatite and collagen) to mimic the bone microenvironment and support the healing process, but all provide substantial drawbacks in clinical application as previously reviewed by Gillman and Jayasuriya.^5^ Nonetheless, approximately 5%–10% of all fractures do not heal completely or only in a compromised manner,^6^ often leading to a scenario of a bone defect that is substantially more challenging to treat. Routine treatment strategies for bone defects include Masquelet technique, Ilizarov technique, the combination of both, or bone grafts/bone graft substitutes used in combination with metal- or ceramic-based endoprosthesis. All of them are associated with advantages and disadvantages as reviewed by Migliorini et al.^7^

To date, to overcome the limitation of clinically-approved bone grafts/bone graft substitutes for bone defect treatment, a variety of novel biomaterials for bone application were suggested, developed, and/or tested.^8^ Montoya et al.^9^ recently revisited the term “smart” biomaterial with regard to its degree of interaction with the bioenvironment. Indeed, the future goal of biomaterials intended to augment bone defect healing should be able to sense the microenvironment, release the appropriate payload (bioactive proteins and small molecules), and adapt their properties as fracture heals and new bone forms. However, the available approved biomolecules are mainly limited to growth factors that may cause severe adverse effects.^5,10^ Additionally, so far, most development of novel biomaterials intended to augment bone healing follow the “fit-all” paradigm neglecting the individual physiological background of patients, especially at diseased conditions. Despite some studies suggesting potential molecular biomarkers that correlate to compromised bone healing capability^11^ or risk of non-union (failure in bone defect healing), none has been identified that is sufficiently understood and clinically confirmed. Molecular profiles of patients may be utilized in clinical decision-making by advising the necessity of biomaterial applications or even guiding personalized biomaterial design that restore the endogenous regeneration capacities.^12^

To address both needs, the identification of bioactive factors and indicative biomarkers, proteomics has proven itself as a powerful tool,^13^ which is capable of characterizing the whole protein content of biological systems both *in vitro* and *in vivo*. It has been successfully applied in bone-related questions to study protein abundance changes,^14^ signaling events,^15^ and protein interactions^16^ as well as for in-depth characterization of bone tissue.^17^ Further, proteomics has recently become a respected approach in clinical decision making.^18^ Given the substantial research undertaken on bone healing, moving toward a coherent framework that collects and synthesizes these disparate data is critical for building a more integrated and full knowledge. Meta-study analysis emerged as a method to capitalize on the vast amount of acquired data. This approach involves systematically compiling and statistically evaluating the findings from a multitude of existing studies harboring the potential to accelerate research at no additional costs for further experiments. Additionally, expanding the pool of observations improves statistical robustness, offering a more powerful way to understand the compiled research. Such an approach surpasses the capabilities of individual studies, positioning it at the top of the hierarchy of evidence.^19,20^ Furthermore, researchers may confirm their single experiments by using aggregated data from meta-analysis. This allows for the reinforcement of findings through cross-validation with a larger variety of data, thus increasing the reliability of their study conclusions. Results from meta-study analyses can be complemented by drug-repurposing approaches. This entails scanning relevant databases for drugs that already target proteins identified through meta-analysis, although for different medicinal purposes. Drug repurposing allows for the use of existing drugs with known safety profiles, significantly reducing development time, cost, and risk.^21,22^

**Figure 1.**
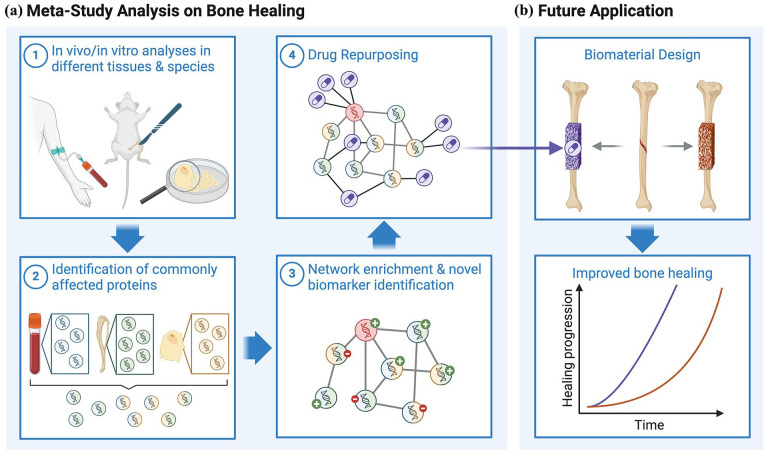
Overview of steps and future application of the meta-study analysis. This meta-study analysis on bone healing (a) comprises four main steps. (1) Studies conducted on different tissues and species were selected according to PRISMA standards. (2) Proteins identified in multiple studies were characterized as commonly affected proteins (CAPs). (3) The identified CAPs were integrated into an existing knowledge base network from NeDRex. This step involved network enrichment by adding necessary interacting proteins, which could serve as novel biomarkers. These proteins were also evaluated for known enhancing or inhibiting effects on bone healing. (4) The proteins of the created network were inspected for known drug targets and their corresponding drugs. (b) The identified proteins and drugs were further evaluated as potential bioactive substances for bone biomaterials to enhance the healing process. Created with BioRender.com.

The present proteomic meta-analysis aims to identify universal molecular features in bone regeneration across different biological hierarchies and species. This will open new opportunities for (i) the extraction of biomarkers that may allow for a clinical prognosis of bone healing quality or that may be utilized as novel endpoints in biomaterial testing, (ii) the linkage of universally affected proteins to biological functions to guide the selection of experimental systems for biomaterial testing and ultimately (iii) the identification of novel biological key players that may be utilized as biomaterial functionalizations or drug targets in future bone graft substitute development. We selected 29 proteomic studies in the context of bone biology, healing, and biomaterial testing spanning *in vitro* and *in vivo* analyses in different species (Figure 1). We extracted commonly affected proteins, integrated known protein-protein as well as protein-drug interactions using NeDRexDB^23^ and Drugst.One^24^ and linked them to biological functions and other external databases to assess their usefulness for the above mentioned objectives. We identified 51 proteins with a potentially high importance in bone regeneration including well-known key components of bone’s extracellular matrix, for example, collagens, fibronectin, and periostin. Further eleven proteins (i.e., ANXA1, HSPG2, and TGFBI) yet out of scope for biomaterial development can be proposed as potential bioactive factors from this meta-analysis. Additionally, we could identify five potential drug targets (i.e., MMP2 and TPM4) and corresponding approved therapeutics for future implant design. The present meta-analysis may not only propose bioactive substances for bone biomaterial development but may also guide testing strategies of novel biomaterials. This meta-analysis connected the identified key proteins to tissue-specific and common interaction partners and functions to select suitable experimental hierarchies and endpoints. Further, we propose ten candidate proteins that could be further investigated as indicative biomarkers to monitor bone healing by liquid-biopsy.

## Results and discussion

### Streamlined meta-analysis workflow in proteomics

**Figure 2.**
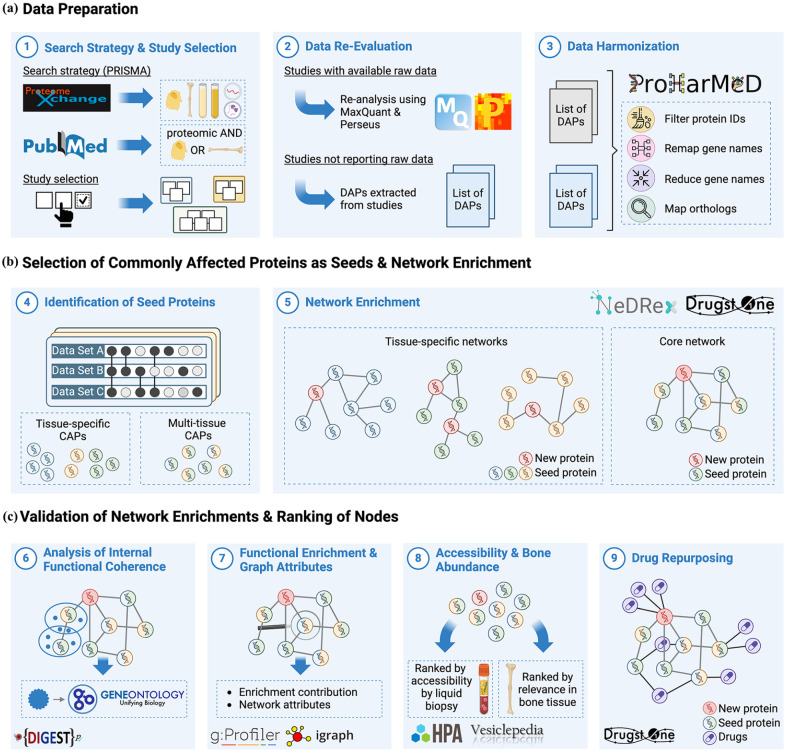
Overview of the analysis steps. (a) In the data preparation phase, the study followed a dual search strategy (1) across ProteomeXchange^25^ and PubMed^26^ adhering to the PRISMA guidelines, to gather osteoblast and bone-related proteomics studies. (2) To ensure comparability, the raw data from these studies were re-analyzed using MaxQuant^27^ with standardized parameters. (3) In the subsequent harmonization step, protein identifiers were unified using ProHarMeD,^28^ aligning them to a common reference species for accurate cross-study comparisons. (b) The next phase involved the selection of commonly affected proteins (CAPs) and network enrichment. (4) CAP selection began with the identification of crucial proteins, those that were consistently present across multiple studies within a single tissue (tissue-specific CAPs) and across multiple tissues (multi-tissue CAPs), using intersection analysis and weighted scoring. (5) These identified CAPs were then enriched into a connected biological network, employing Drugst.One^24^ and NeDRexDB^23^ to uncover potential protein-protein interactions. (c) Lastly, the validation and ranking of nodes was performed. (6) It involved analyzing the internal functional coherence of gene subnetworks using DIGEST,^29^ which assessed the significance and contributions of individual proteins. (7) This was complemented by functional enrichment analysis with g:GOSt^30^ and the evaluation of graph attributes using the igraph library.^31^ Genes were then ranked based on enrichment scores and their roles in the network topology (centrality measures). (8) Additionally, proteins were ranked for their accessibility via liquid-biopsy and relevance in bone tissue using data from the Human Protein Atlas^32^ and Vesiclepedia.^33^ (9) The last step was drug repurposing, where Drugst.One was utilized to explore the network’s proteins for their drug targeting potential and to identify key drugs for potential repurposing. Created with BioRender.com.

To make use of the wealth of information contained within publicly available datasets, we developed a comprehensive workflow (Figure 2). It was designed to span the whole spectrum of data utilization ranging from data preparation to advanced data analysis including network enrichment and validation of the results. In the data preparation phase of the project, proteomic data from bone-related studies at *in vitro* and *in vivo* level were gathered from ProteomeXchange^25^ and PubMed,^26^ re-analyzed using MaxQuant^27^ for standardized comparison, and proteins were unified using ProHarMeD^28^ for robust cross-study comparisons. The next phase involved the selection of proteins, which were identified as differential abundant across different studies (commonly affected proteins, CAPs) by intersection analysis and weighted scoring. Subsequently, network tools such as Drugst.One^24^ and NeDRexDB^23^ were utilized to find significant protein-protein relationships and enriched functions in biological networks built from CAPs (seed proteins) and connecting proteins (exception proteins). The final phase involved evaluating and ranking nodes within the network using DIGEST^29^ to assess gene subnetworks’ internal coherence, functional enrichment analysis, and mapping of biological knowledge bases to determine the biological relevance, gene network centrality to measure the importance inside the network, and Drugst.One to investigate drug repurposing opportunities. The Methods part of this publication provides more thorough descriptions of the approaches employed.

### Overview of collected studies and discovery of commonly affected proteins within single tissues

**Figure 3.**
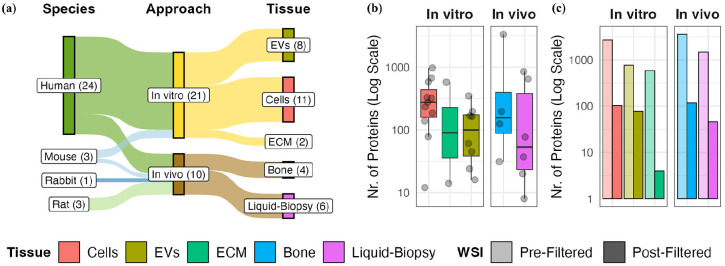
Summary of included studies and selection of co-affected proteins for further analysis. (a) Overview of research background of included studies including species for specimen origin, approach, as well as tissue type. Box sizes represent the number of studies associated with objects, which is additionally indicated in brackets. (b) Number of extracted differential abundant proteins (DAPs) per study and tissue type. Each dot represents a single study. Whisker plot represents the distribution of DAPs per tissue type with 25–75th percentile and median value. (c) Tissue-specific commonly affected proteins (CAPs) were identified by the weighted scoring intersection (WSI) method for each tissue type. Bar heights represent the number of unique cumulative DAPs prior to and selected tissue-specific CAPs after WSI filtering.

To generate a global map of molecular effects in bone regeneration and healing, 31 datasets from 29 proteomics studies^34–62^ were selected representing four species and different testing approaches, biological matrices as well as hierarchies (Supplemental Table 1, Figure 3(a)). The majority of datasets (*N* = 21) represent *in vitro* analyses of cells, cell-derived extracellular vesicles (EVs) or ECM with 19 being performed with cells of human origin and two of murine origin. All studies applying analysis of regenerating bone tissue (*N* = 4) originate from animal studies including rat and rabbit. The remaining datasets (*N* = 6) originate from human clinical or murine preclinical samples and focus on biomarker research and liquid- biopsy-accessible proteins with four representing direct analysis of soluble plasma/serum proteins and two of serum-circulating EVs.

To identify CAPs, differentially abundant proteins (DAPs) were extracted from the selected studies by re-analyzing available raw data or from published lists in original research articles (see Method section, Supplemental Table 1). We utilized ProHarMeD, a tool for harmonizing and comparing proteomics data across multiple studies, which enables ID conversions, disease mechanism extraction, and drug repurposing suggestions. This allowed us to standardize and compare these DAPs effectively across different protein databases and species used for single analysis (Supplemental Figures 1 and 2). Upon harmonization, we obtained DAPs specific to each tissue type. Depending on the specific study, the total numbers of DAPs differs significantly (Figure 3(b), Supplemental Table 1), with medians of 276 DAPs in *in vitro* cellular assays, 112 in *in vitro* EV analyses, 296 in *in vitro* extracellular matrix (ECM) analyses, 161 in regenerating bone analyses, and 57 in biomarker screenings in plasma/serum and circulating EVs. Next, the Weighted Scoring Intersection (WSI) approach was applied to the DAPs of each tissue type independently to identify proteins with high re-identification rates, called tissue-specific CAPs. The WSI approach calculates a weighted score for each protein based on its presence in multiple studies, adjusting for tissue-specific biases and varying study sizes (Supplemental Figure 3). For more details on the study selection, data harmonization, and DAP extraction processes, please refer to the *Study Selection, Data Re-evaluation for Proteomics Raw Data* and *Retrieval of Differentially Abundant Proteins and Meta-Study Harmonization* sections in the methods. The detailed explanation of the WSI approach, including the scoring calculations and criteria for inclusion, can be found in the *Selection of Commonly Affected Proteins* section of the methods. By applying this approach, we allow the inclusion of proteins that would be lost by simple filtering based on the presence in at least half of the available studies per tissue. Consequently, we identified 4–113 tissue-specific CAPs, which were further utilized as seed proteins in our network construction (Figure 3(c)).

Analyzing the IDs before and after harmonization highlights the crucial importance of the harmonization process. This step enhanced the intersection size of 110 proteins out of 322 that were identified as significant after the weighted scoring intersection. More importantly, for 95 of those proteins, the intersection size increased from 1 to 2, allowing them to remain viable candidates (Supplemental Figure 4). Without this harmonization step, those proteins would have been excluded due to the cut-off which ignores proteins occurring only once across the studies.

### Unveiling tissue-specific networks and functional annotations

To identify potentially important proteins and their functions across various tissues, we employed the previously identified tissue-specific CAPs as seed proteins for a network enrichment analysis. Therefore, the tissue-specific CAPs were first mapped onto a comprehensive protein-protein interaction (PPI) network, sourced from NeDRexDB,^23^ which is a comprehensive knowledge base combining information from multiple databases. We chose NeDRexDB based on a comparative evaluation of the coverage of the identified CAPs in commonly used sources like IID,^63^ STRING,^64^ and BioGRID^65^ to NeDRexDB. Our evaluation revealed that NeDRexDB, which contains the highest number of covered IDs and interactions, provides the best coverage for all tissues (Supplemental Figures 5 and 6). Using the Multi-Steiner Tree algorithm,^66^ tissue-specific CAPs were then integrated into a single connected network separately, adding a minimal amount of exception proteins to ensure connection among all seed proteins. This process resulted in significantly functional coherent networks for each tissue type, which are the tissue-specific networks (Table 1, Supplemental Figures 7–11, Supplemental Table 2). The added exception proteins were divided into three categories: those that appeared as CAPs in another tissue after WSI filtering (“CAP in other tissue”), proteins that were listed as DAPs in a study but eliminated during WSI (“study-specific DAP”), and novel proteins that had not been reported as DAPs in any of the selected studies (“no DAP”). Both seed and exception proteins of the single tissue-specific networks were considered for all subsequent downstream analyses.

**Table 1.** Single tissue-specific network sizes.

Tissue	Number of tissue-specific CAPs (seeds)	Number of added proteins (exception nodes)		
		CAP in other tissue	Study-specific DAP	No DAP
Cells	101	1	4	9
EVs	77	0	8	9
ECM	4	0	1	3
Bone	113	2	9	11
Liquid-biopsy	31	1	11	6

The number of commonly affected proteins (CAPs) refers to the number of proteins after undergoing weighted scoring intersection (WSI) filtering that were used as seeds in network enrichment to create a single connected network for each tissue type. The number of added proteins, that is, exception nodes, necessary to connect the seeds was further separated into three categories: proteins that meet the criteria for CAPs in another tissue after WSI filtering (CAP in other tissue), proteins that were listed as a differentially abundant protein (DAP) in any of the datasets included in the meta-analysis but eliminated during WSI filtering (study-specific DAP), and novel proteins that have not been reported as DAP in any of the selected studies (no DAP).

To analyze which biological functions are covered by the constructed networks, functional enrichment analyses were conducted for each single-tissue network, resulting in a total of 643 enriched terms across all included tissue types. However, of these terms, a majority of 462 were exclusively associated with one specific tissue type (Supplemental Table 3). For instance, *in vitro* cell-based assays cover proteins associated to general housekeeping mechanisms like RNA binding, ribosome assembly and translation elongation (40S and 60S ribosomal proteins as well as eukaryotic translation elongation factors) as well as protein processing (AHSG, CCN1, SERPINH1, PLG, and TGM2), organelle organization (VMP1 and ITPA), adherens junctions (annexins), and chemoattractant activity (FGF2 and S100A4). *In vitro* EV-based studies provide the most specifically enriched functions including proteins that regulate cell migration, ECM binding and assembly, cell-matrix interaction, cartilage development as well as orchestration of wound healing (integrins, collagens, CCN1/2, POSTN, LTBP2, FLNA, LRRC15, and ABI3). Additionally, proteins involved in TGF-beta production and binding were enriched among EV-focused analyses (FN1, LTBP1, and THBS1). In contrast, proteomic analyses of ECM provide the least specifically enriched functions, including proteins that are linked to calcium-mediated signaling (CD4, SLC8A1, and TRPM4) and cell adhesion (ANXA1 and S100A11). *In vivo* analysis of regenerated bone provides enriched proteins involved in molecular processes related to proteolysis and protein maturing (AHSG, HTRA1, SERPINA1, and SERPINC1), but also apoptotic processes (CTBS, ANXA1, PITX3, KNG1, and GPLD1) and wound healing (SPP1, fibrinogen, PRDX2, and STAT3). Furthermore, proteins involved in the interaction with other tissues, for example, muscle system (ACTA2, CALD1, DYSF, and NEB) and vascular system (ADIPOQ, FABP5, and YWHAE) can be covered by direct analysis of regenerated bone. Liquid-biopsy studies in the context of biomarker identification specifically cover proteins related to cytokine production and inflammatory response (IL1B and CXCL8). Thus, the majority of observed effects are exclusively addressable in specific tissues. In the development of innovative biomaterials designed to enhance bone regeneration, selecting the appropriate target tissue types for analysis—such as cells, extracellular vesicles (EVs), extracellular matrix (ECM), bone, and liquid-biopsies-is crucial. The choice should align with the novel biomaterials’ anticipated biological functions to ensure the most effective outcomes.

### Common functional enrichments across study types

**Figure 4.**
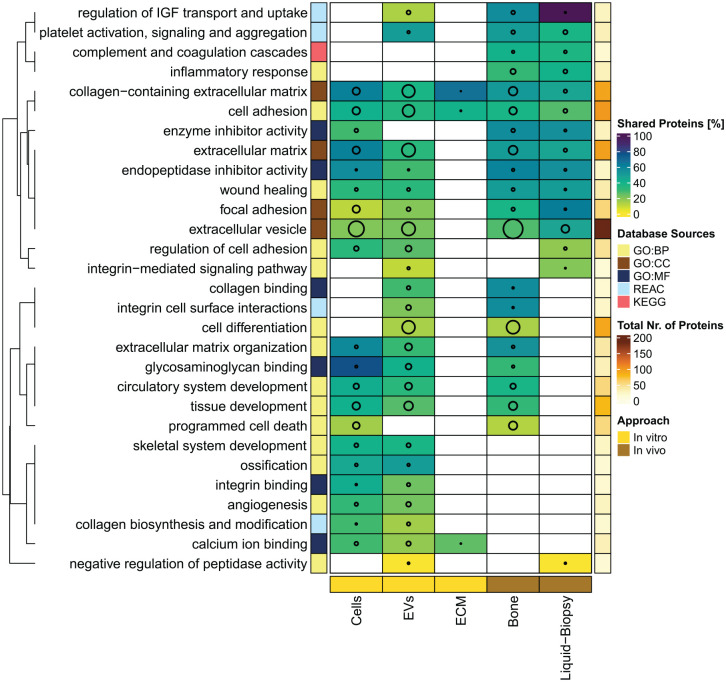
Selection of shared enriched functions across tissue types. Twenty-nine functions were manually selected from all 181 enriched terms (Supplemental Table 3) that were shared across at least two tissue types. Heatmap represents the number of proteins per tissue type assigned to selected functions (circle sizes) and the proportion of proteins that are shared with at least one other tissue type (color scale). The left annotation represents the database for term retrieval (color code Database Sources), either gene ontology (GO), Reactome (REAC), or KEGG Pathway database (KEGG). Right annotation represents cumulative numbers of assigned proteins across all tissue types for each selected function. Clustering and dendrogram represent the Jaccard distance of selected functions.

To identify molecular key mechanisms that are affected across different biological hierarchies and species, 181 common functions that are shared between two or more tissue types were extracted from the functional enrichments of single-tissue networks (Figure 4, Supplemental Table 3). Proteins involved in cell adhesion (e.g., ANXA1, FBN1, and LGALS1) or being components of collagen-containing extracellular matrix (COL1A1, FN1, and POSTN) were enriched among CAPs in all tissue types. Both are main functions that need to be fulfilled for proper bone regeneration. Thus, innovative biomaterials for bone application aim for osteoconductivity or mimic the natural collagen-containing bone ECM.^67,68^ Proteins known to be associated with EVs (AHSG, CTSA, and S100A4), involved in wound healing (ANXA1, PLG, and KNG1), focal adhesion (NPM1, HSPG2, and YWHAE), or possessing an endopeptidase inhibitor activity (SERPINA1, SERPINC1, and SERPINH1) were enriched in four tissue types only excluding ECM-focused analysis. EVs are both acknowledged as intercellular mediators and known transporters between osteoblasts and bone ECM.^69^ By this, EVs mediate wound healing circuits, osteoimmunity, and provide focal adhesion sites.^70,71^ Calcium ion binding proteins involved in mineralization of bone were enriched in all three *in vitro* but none *in vivo* tissue types (annexins, LTBP2, and THBS2). Proteins associated with tissue development and circulating system development (CCN1/2, LMNA, and TGFBI), as well as ECM organization and glycosaminoglycan binding (CCDC80, LTBP2, and POSTN) were covered in *in vivo* bone regeneration studies but could also be observed by cell- and EV-focused *in vitro* approaches. Glycosaminoglycans possess a pivotal role in bone hemostasis and modulate angiogenesis, ECM organization and bone tissue development.^72,73^ Platelet activation, signaling, and aggregation, as well as regulation of IGF transport and uptake (AHSG, APOA1, FN1, and C3) were enriched among proteins co-affected in *in vivo* bone regeneration studies and liquid-biopsy studies. IGF1 is involved in bone remodeling and its transport and uptake by IGF-binding proteins modulates its action.^74^ Interestingly, those functions were also enriched in *in vitro* EV-focused studies, which may indicate to directly connect *in vitro* EV-focused studies to *in vivo* studies. Proteins associated with the integrin-mediated signaling (ITGA3, ITGA5, ITGAV, and ITGB5) and negative regulation of peptidase activity (CD44, LRP1, and A2M) were enriched in liquid-biopsy studies and *in vitro* EV-focused studies. Integrins contribute to cell adhesion and vascularization in bone and novel biomaterials aim to provide integrin binding sites to increase bone regeneration.^75^ Among co-affected proteins in bone regeneration studies, collagen-binding proteins (CTSB, CTSS, and THBS1), proteins involved in cell differentiation (ABI3, STAT3, and POSTN) and integrin cell surface interactions (SPP1, CD44, and collagens) were likewise enriched in EV-focused *in vitro* studies. Note that, several key functions in bone regeneration could only be analyzed in *in vitro* studies, for example, skeletal system development, ossification, collagen synthesis, and modification (BMP1, CCN1/2, and FGF2) as well as angiogenesis (CALD1, DCN, and LOXL2). In contrast, proteins in inflammatory response (SOD1, IL1B, and CXCL8) as well as complement and coagulation cascades (C1QA, C3, and C4A) were only enriched in *in vivo* studies.

Thus, this meta-analysis provides an overview, which key functions of bone regeneration can be covered by different experimental models. This allows the selection of suitable test systems for the evaluation of innovative biomaterials that target certain functions. Interestingly, although similar functions were enriched across different study types, their observations are based on differently involved proteins. For instance, less than half of all 100 CAPs associated with cell adhesion were re-identified between two or more study types (Figure 4). Thus, each tissue type covers certain subclusters contributing to cell adhesion (Supplemental Table 4). Similar observations were gained for proteins annotated as collagen-containing ECM components (Supplemental Table 4).

Thus, only a minority of biological functions (181 out of 643) can be monitored in different tissue types. Only eight functions can be covered in four or five tissues. Interestingly, EVs appear to link functions that are otherwise restricted to *in vivo* analysis or to *in vitro* studies.

### Core network extraction and protein prioritization

**Figure 5.**
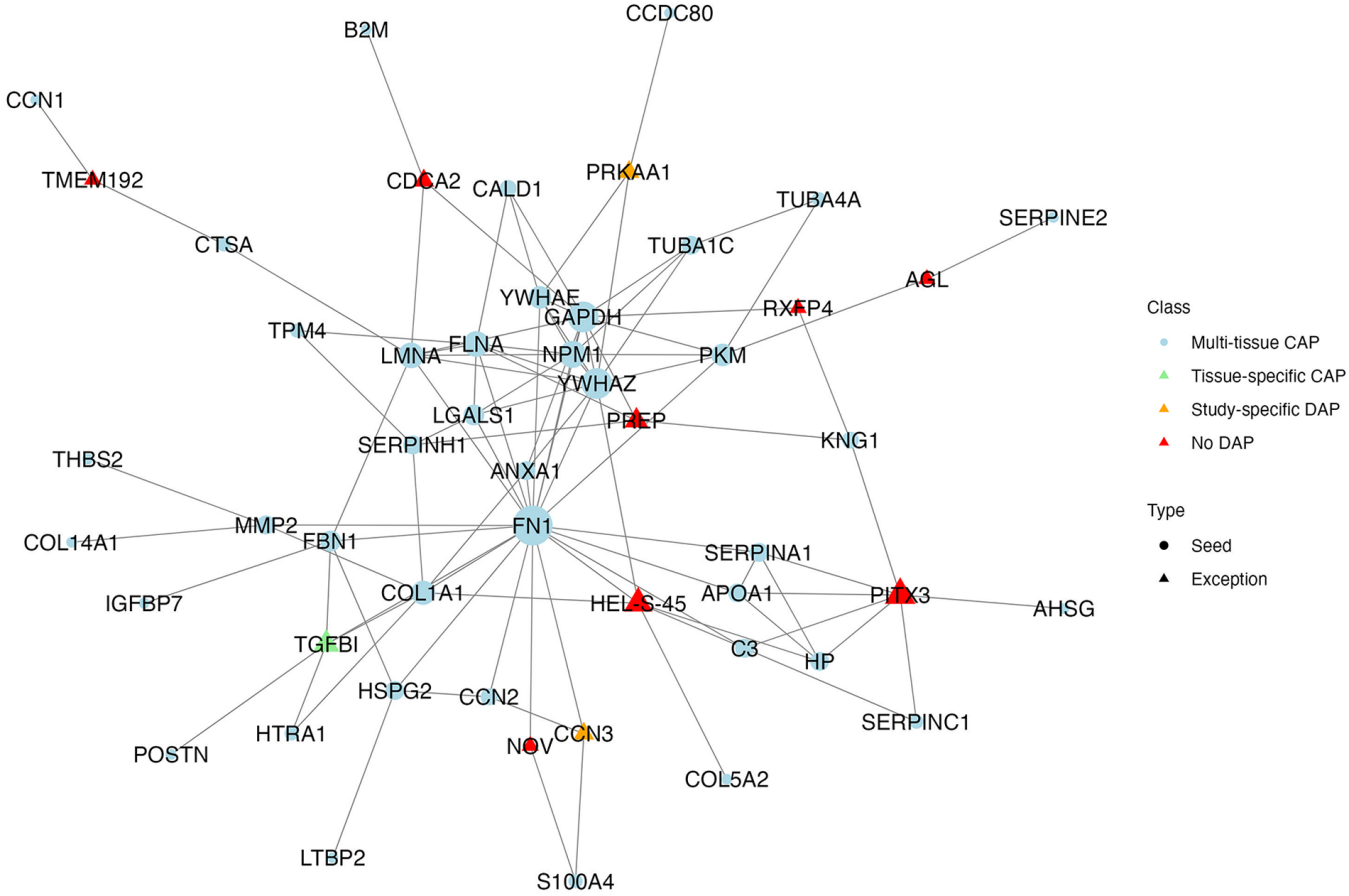
Enriched core network of shared commonly affected single tissue proteins. A total of 40 multi-tissue commonly affected proteins (CAPs), here indicated as seed, were mapped into the NeDRexDB^23^ network and connected with the Multi-Steiner Tree algorithm to create a single connected component by adding 11 exception proteins. The 11 exception proteins were further assigned to three subcategories: Proteins that were commonly affected in only one single tissue and not across tissues (tissue-specific CAP), proteins listed as differentially abundant protein (DAP) in a study but not identified as commonly affected (study-specific DAP), and proteins that were not listed as DAP in any of the initial studies (no DAP).

When we examined the common biological processes, we noticed that while the same functions were shown to be enriched across different tissues, these functions were driven by distinct sets of proteins in each tissue type. Despite this variety, a group of proteins was evident as key participants in many tissues. Proteins that are affected in different tissues are of particular interest to our study because they may serve as multifunctional candidate proteins. Therefore, this part of the analysis focused on only including the 40 CAPs that were identified in more than one single tissue, referred to as multi-tissue CAPs. Those 40 proteins were used as seeds for constructing a core network that potentially highlights intricate multi-tissue interactions. Thus, proteins in this network can be considered as key proteins integrating bone regeneration-relevant mechanisms across different biological hierarchies and will be focused in all following evaluation steps. This reconstructed network includes 11 additional proteins, that is, exception nodes: TGFBI already identified as significant in a single tissue, two (CCN3 and PRKAA1) that were initially filtered out but re-emerged in the analysis, and eight new proteins (PITX3, HEL-S-45, PREP, NOV, AGL, CDCA2, RXFP4, and TMEM192) not previously identified (Figure 5, Supplemental Table 2).

TMEM192 was previously added as an exception node to the single tissue network of cells. Its role there is to act as a connector for CCN1, a function it also fulfills in the core network. Additionally, HEL-S-45 and PRKAA1 appeared as exception nodes in the EVs single tissue network. Notably, PRKAA1 appeared as DAP in studies related to tissue categories Bone and Liquid-biopsy but was subsequently excluded due to the WSI filtering thresholds. In the core network, PRKAA1 was introduced as an exception node specifically to connect CCDC80, a seed protein identified in both Cells and EVs. This inclusion of PRKAA1, despite its earlier exclusion due to WSI filtering thresholds, underscores its potential relevance in linking key proteins across different tissues.

**Figure 6.**
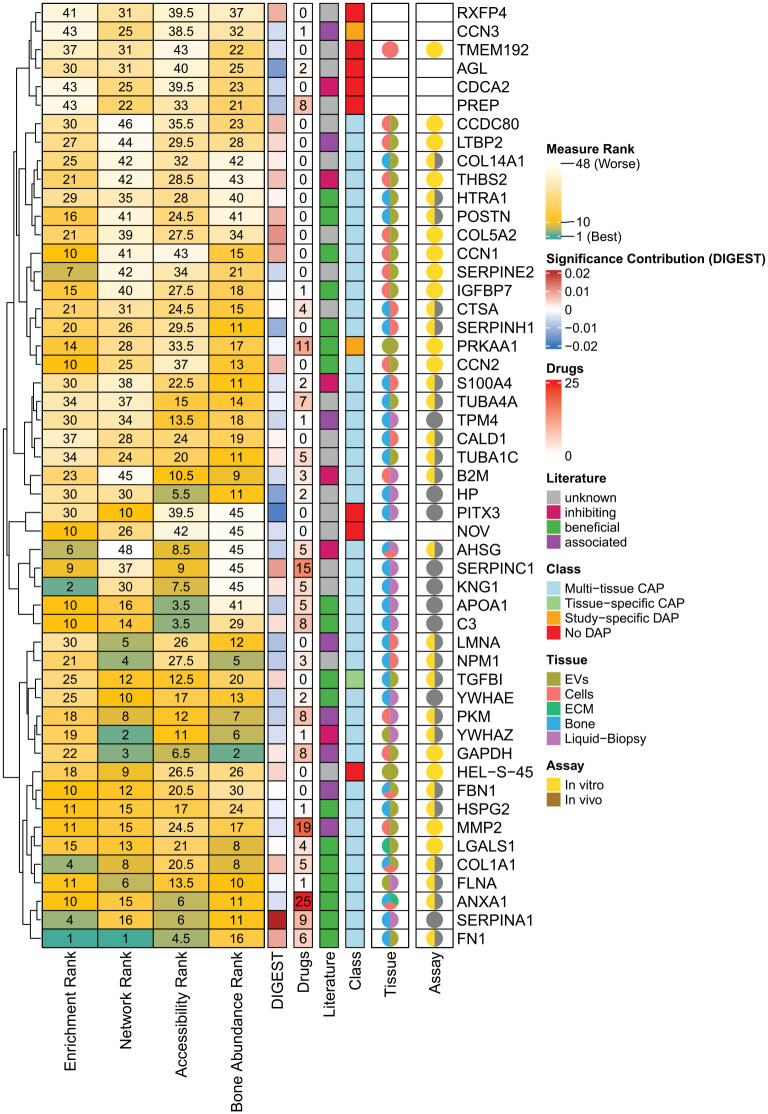
Enhanced scoring and analysis of core network proteins. This comprehensive heatmap shows the median rank of each protein across numerous sub-rankings. The heatmap is enhanced with additional row annotations. The first annotation, “DIGEST,” displays the median significant contribution of each gene based on common Gene Ontology (GO) keywords and KEGG pathway terms. The “Drugs” annotates the number of chemicals or medications that have been identified to target each gene. The “Literature” annotation describes the gene’s effect on bone healing as assessed by relevant literature. Class assigns each protein to one of four categories: a multi-tissue commonly affected protein (CAP) that was used as a seed node in network enrichment, a protein that was commonly affected in only one single tissue and not across tissues (tissue-specific CAP), a protein listed as differentially abundant protein (DAP) in a study but not identified as CAP (study-specific DAP), or a protein that was not listed as DAP in any of the initial studies (no DAP). Finally, the “Tissue” annotation displays a pie chart for each gene, indicating their presence as nodes in the tissue-specific networks. The row-wise clustering of the heatmap is based on the Jaccard index calculated from the ranking values.

The main goal of the meta-analysis is to identify proteins that have not only cross-tissue significance but also provide further features that make them ideal candidates as biomarkers, bioactive proteins for biomaterial functionalization or potential drug targets. Therefore, we applied different scoring metrics and integrated external knowledge bases as described in the method section. The 51 proteins, that is, 40 seed and 11 exception proteins, of the core network were ranked according to these scores. These rankings include (Figure 6, Supplemental Table 5):

- Enrichment rank: Measures how significantly protein functions, like biological processes or molecular functions, are overrepresented in a gene list.
- Network rank: Evaluates a gene’s role and importance in the network topology using metrics like centrality and connections.
- Accessibility rank: Includes measured abundance of proteins in blood plasma and previous knowledge about secretion status as well as participation in EV cargo retrieved from public databases (Human Protein Atlas (HPA) and Vesiclepedia). This reflects the likelihood of protein accessibility in liquid-biopsies
- Bone abundance rank: Reflects the likelihood of proteins to be a vital part of bone tissue by including measured expression in bone and bone-associated cells as well as secretion status to ECM, retrieved from external database HPA.

The ranking was accompanied by functional contribution scores determined by DIGEST, which evaluates a protein’s contribution to the overall functional coherence of the core network using the mean Jaccard index across protein pairings. In addition, 30 of the 51 core proteins were identified as drug targets with associated drugs specifically targeting them (Figure 6, Supplemental Table 5).

High rankings in Network and Enrichment Rank and high DIGEST score indicate highly connected proteins with functional relevance. Thus, these metrics aid in prioritizing proteins for later selection as multifunctional candidates. TGFBI, FN1, and C3 stand out in our study due to their top ranking based on network connectivity. Although TGFBI does not possess an enrichment rank as high as FN1 and C3, it still plays an important part in biological processes associated with the core network, according to DIGEST. PRKAA1 and HSPG2 exhibit significant enrichment scores in the core network and POSTN improves the network’s functional coherence. ANXA1, FLNA, LGALS1, COL1A1, FBN1, and MMP2 all have high network connectivity and top enrichment rankings, indicating that they play an important role in the network’s biological activities. APOA1, AHSG, KNG, and SERPINC1 are vital to the network’s biological process. Thus, assessing the network’s topology and functional relationships elucidated central hub proteins of importance for the core network.

### Identification of potential biomarkers

To be considered as a potential biomarker to monitor bone healing, candidate proteins should either be directly linked or functionally related to bone healing mechanisms and need to be accessible via easy sampling techniques, for example, plasma, serum, or circulating EVs. Thus, we added the accessibility rank and bone abundance rank to our evaluation of the 51 core proteins.

TGFBI, FN1, HTRA1, POSTN, and HSPG2 were affected in studies in healing bone *in vivo* and in *in vitro* sampled EVs. EVs are thought to have an important role in promoting communication between bone cells.^69^ Thus, proteins transported via EVs, reaching the circulation and that can be sampled via liquid-biopsy may be candidates to gain insights into bone homeostasis by indirect sampling.^71^ In particular, FN1, TGFBI, and HSPG2 possess high accessibility ranks. All five proteins are either directly connected, for example, TGFBI connects POSTN to HTRA1, or are in a proximity of one connecting node in the network, indicating a functional relationship between these proteins. Additionally, all five proteins were previously related to bone formation or regeneration. POSTN stimulates the migration, adhesion, and proliferation of human adipose tissue-derived mesenchymal stem cells.^76^ Moreover, it has previously been proposed as a liquid- biopsy-accessible biomarker of osteoporosis in postmenopausal women with diabetes and in general fracture healing response.^77,78^ TGFBI is associated with building bone mass and strength *in vivo*.^79^ It was reported to be expressionally upregulated in cartilage and bone but downregulated in hMSC of osteoarthritic patients.^80^ Still, we are the first to suggest TGFBI as a potential liquid-biopsy-accessible biomarker. Similarly, HSPG2 was found to modulate the BMP2-mediated osteogenesis and its longitudinal expression in fracture healing *in vivo* correlates with other healing markers.^81,82^ However, it was not proposed as a liquid-biopsy-accessible biomarker before. FN1 is a main component of bone ECM and acts as a three-dimensional linker within it.^83^ Besides that, it has been described as a potential biomarker in various diseases including tumors, aortic valve calcification, and diabetic neuropathy but not yet in bone regeneration.^84–86^ HTRA1 is localized predominantly to areas of new bone formation during bone fracture repair, implicating an increased abundance during proper bone regeneration.^87^ However, we are the first who propose this protein as a potential bone healing biomarker. Due to their proven evidence to be responsive in bone healing studies *in vivo* and in EVs as well as previously described functional relationship to bone healing mechanisms, TGFBI, FN1, HTRA1, POSTN, and HSPG2 could be considered as potential biomarkers in bone healing studies. However, within this meta-analysis, they lack evidence for being affected in liquid-biopsy studies. Future studies need to prove their potential as indicative biomarkers.

In contrast, YWHAE, HP, TPM4, APOA1, and AHSG are prevalent and affected in both *in vivo* bone healing studies and liquid-biopsies in this meta-analysis. Additionally, they provide high accessibility ranks, anticipating an easy sampling and potential usage as indicative biomarkers in bone healing studies. In particular, APOA1 and AHSG are additionally vital to the network’s biological process. AHSG is mainly produced by osteocytes in bone and regulates postnatal bone growth and remodeling.^88,89^ Additionally, it was previously suggested to monitor scaffold-guided regeneration in animal models and bone mineral density variation.^90,91^ However, these suggestions were based on genetic evidence or at transcript levels after tissue explantation and not liquid-biopsy. APOA1 was previously suggested as a biomarker of several diseases, for instance aplastic anemia, atherosclerosis, thrombosis, diabetes, cancer, and neurological disorders.^92,93^ Although it was shown to regulate osteoblast precursor cells,^94^ we are the first who postulate it as a potential biomarker in bone healing. Similarly, YWHAE was shown to enhance the osteogenic differentiation^95^ whereas TPM4 regulates adhesion structures and resorptive capacity in osteoclasts.^96^ Both have not been suggested as bone-healing biomarkers before. HP was shown to be related to acute phase response after osteotomy and heterotropic ossification.^97,98^ Thus, utilization as a biomarker in bone regeneration might be possible.

In conclusion, our thorough approach to assess the core network and its topology, integrating the evidence in which tissue types proteins were affected alongside external knowledge bases and literature research, lead to the identification of biomarker candidate proteins, namely TGFBI, FN1, HTRA1, POSTN, HSPG2, HP, APOA1, AHSG, TPM4, and YWHAE. For all of them but POSTN we are the first to suggest their bone biomarker potential.

### Potential bioactive proteins for scaffold functionalization

In addition to their usability as bone-healing indicating biomarkers, the identified 51 core proteins may be exploited as potential candidates for functionalizations of bone biomaterials to support bone healing processes. To be considered as candidates, proteins were selected from the 51 core proteins by possessing high bone abundance ranks and to be previously shown to enhance bone regeneration.

Among those candidates are highly abundant structural components of the bone ECM that were extensively studied in bone biology and previously utilized in biomaterials intended for bone healing, for example, COL1A1 and FN1.^99,100^ Additionally, also less extensively studied proteins proven to enhance bone formation *in vivo* were identified by the presented approach. POSTN and YWHAE were both previously shown to positively stimulate osteogenesis when loaded into scaffolds.^76,95^ Further, HSPG2 is involved in bone calcium signaling and was shown to accelerate vascular development and BMP delivery when utilized to functionalize biomaterials.^101–103^ This evidence proves the selected streamlined meta-analysis workflow to extract functional players in bone regeneration, that have previously been successfully tested as biomaterial functionalization. Beyond that, this meta-analysis suggests novel biomaterial functionalization candidates to augment bone healing that have not been tested as implant coatings previously. As such, CCN1 has a high bone abundance rank and was shown to enhance osteoblast differentiation and bone mineralization of periodontal ligament stem cells *in vitro* as well as the proangiogenesis.^104,105^ Indeed, the potential to induce capillary invasion of CCN1-modified hydrogels were assessed in chorioallantoic membrane assays.^106^ However, no application for bone biomaterials was yet conducted. HTRA1 is secreted by MSC upon osteogenic induction and itself positively regulates osteogenesis *in vitro*. In healing bone it is mainly localized in the regenerated tissue.^87^ However, the exact role of HTRA1 in bone healing remains a mystery. Loss-of-function studies revealed a contradicting role of HTRA1 *in vitro* and *in vivo*.^107^ Moreover, it is reported to be involved in the development of skeletal disorders as reviewed by Tossett et al.^108^ Thus, thorough validation of the utilization of HTRA1 as a bioactive component in innovative bone biomaterials need to be applied. IGFBP7 both stimulates the osteogenic differentiation of MSC and reduces osteoclastogenesis.^109,110^ Moreover, in a murine bone fracture model, IGFBP7 accelerates the bone healing when administered intravenously especially in combination with the parathyroid hormone.^111^ However, it was not tested as a biomaterial functionalization before. LGALS1 induces osteoblast differentiation *in vitro* as well as *in vivo*^112,113^ and reduced serum as well as tissue abundance levels correlated to age-related bone mass loss.^114,115^ TGFBI was previously shown to affect skeletal tissue maintenance by modulating the ECM degradation in mice.^116^ Both LGALS1 and TGFBI have not been tested in bone biomaterials before. Thus, the proteins CCN1, HTRA1, IGFBP7, LGALS1, and TGFBI are all known bone healing-supporting components of bone ECM and thus, novel potential candidates for biomaterial functionalizations.

However, other proteins than classical ECM components such as secreted proteins might also be considered as potential bone biomaterial functionalizations. For instance, osteoclast-derived C3a stimulates osteoblast differentiation.^117^ Further, C3-deficiency negatively affects fracture healing.^118^ ANXA1 is released as a component of EVs and these ANXA1-containing EVs activate wound repair circuits.^70^ Indeed, injectable ANXA1-modified hydrogels were shown to accelerate regeneration processes.^119^ However, these findings were limited to murine dorsal wound models and no studies towards bone regeneration were conducted. SERPINA1 possesses anti-inflammatory properties and were shown to increase the proliferation of MSC.^120^ In a diabetic mouse model of impaired wound healing, SERPINA1-loaded EVs were found to promote tissue repair.^121^ Moreover, direct intraperitoneal injection of SERPINA1 or transplantation of genetically engineered SERPINA1-overexpressing adipose tissue-derived mesenchymal stem cells reduces bone loss in osteoporotic mice models.^122,123^ However, direct release of SERPINA1 from biomaterials for bone regeneration have not been tested before.

All above-mentioned proteins were enriched in the core network of the presented meta-analysis indicating their role as key proteins across different biological hierarchies. This is accompanied by high rankings in the bone abundance scores (Figure 6) as well as supporting evidence from external literature not included in this meta-analysis. This meta-analysis further supports the evidence of COL1A1, FN1, POSTN, YWHAE, and HSPG2 to be promising candidates in the functionalization of bone biomaterials. All five proteins have already been tested successfully. Moreover, with CCN1, HTRA1, IGFBP7, LGALS1, TGFBI, C3, SERPINA1, and ANXA1 we propose eight novel bioactive candidates for bone biomaterial innovations.

### Potential drug targets and therapeutics

A third strategy to exploit the identified 51 key proteins is to identify novel drug targets by a repurposing strategy. In this meta-analysis our main focus is on drug targets that have an inhibitory role in bone regenerative mechanisms. These target proteins may be addressed by small molecule therapeutics that could act as antagonists on the undesired outcomes, and thus enhance bone regeneration. However, we also included drugs that target multiple proteins regardless of their beneficial or inhibitory role. We further included external references to select drugs with known bone regeneration enhancing effects.

A total of 133 drugs were identified to target 30 of the 51 identified core proteins (Supplemental Table 5). Most identified drugs target 1–2 proteins, only 7 out of 133 target multiple proteins in the core network. Six out of those seven are broad-acting, including four metal ion-based molecules, such as Zinc salts. The other of those drugs is artenimol, an approved anti-Malaria drug,^124^ which targets the core proteins NPM1, PKM, GAPDH, FLNA, and LGALS1. Although none of the targeted proteins was directly associated with bone healing before, a recent study shows that artenimol promotes osteogenesis and can effectively reverse osteoporosis-related bone loss.^125^ It was shown to promote osteoclast apoptosis and suppress RANKL-induced osteoclast differentiation in different mouse models.^126–129^ Thus, artenimol may be a suitable candidate to treat bone loss diseases like osteoporosis, but clinical trials are still pending. As a more direct evidence, trifluoperazine—an antipsychotic drug approved for clinical use—targets S100A4.^130^ S100A4 itself inhibits the osteoblast function *in vitro* and *ex vivo* at least partly by activating NFΚB signaling.^131^ A S100A4 knockdown was further shown to enhance the expression of osteoblast marker genes. Thus, targeting S100A4 by trifluoperazine may be an option to increase bone regeneration. Additionally to its S100A4-targeting mechanism, trifluoperazine was also found to reduce osteoclastogenesis by inhibiting calmodulin signaling.^132^ Besides this evidence making trifluoperazine a candidate worth considering, thorough validation needs to be applied as oral application of antipsychotic drugs like trifluoperazine were previously associated with bone mass loss in humans.^133^ Phenethyl isothiocyanate—a naturally occurring metabolite from cruciferous vegetables tested for anti-cancer therapies^134^—was found to target four of the identified core proteins including YWHAZ. Knockdown experiments of YWHAZ showed to enhance osteoblastogenesis and mineralization *in vitro* as well as to increase bone strength in a osteoporotic mouse model.^135^ Phenethyl isothiocyanate additionally targets TPM4, which itself binds and stabilizes actin microfilaments. A knockdown of TPM4 was shown to reduce the adhesion and resorptive activity of osteoclasts.^96^ Thus, targeting YWHAZ and TPM4 by phenethyl isothiocyanate may increase bone regeneration by a dual mechanism which may be especially worth considering in diseases related to increased bone resorption. This is further supported by evidence of a phenethyl isothiocyanate-induced suppression of osteoclastogenesis by blocking RANKL-mediated signaling.^136^ Nineteen drugs were found to target MMP2 (Figure 6). MMP2 is a major regulator in bone healing and MMP2-mediated effects can vary depending on the context.^137^ Both excessive and insufficient MMP2 activity can contribute to inadequate bone repair. However, inhibition of MMP2 was proven to promote osteogenesis and angiogenesis *in vitro* as well as bone repair *in vivo*.^138^ Quercetin is one of the small molecules to target MMP2. Studies have demonstrated that quercetin shows promising therapeutic effects on bone health, including the ability to protect human osteoblasts from cigarette smoke-induced damage, effectiveness in managing diabetic osteopenia, as seen in diabetic rats, and its protective role against high glucose-induced damage.^139–142^

Thus, in addition to the above-mentioned prospective bioactive candidates for the functionalization of biomaterials, the presented meta-analysis suggests trifluoperazine, phenethyl isothiocyanate, quercetin and artenimol as prospective drug candidates to enhance bone regeneration. All of them have previously proven to maintain or restore bone functions *in vivo*, especially at diseased conditions like osteoporosis. However, larger (pre-)clinical studies need to validate the here collected findings. In addition, by applying protein/drug-interaction networks, S100A4, YWHAZ, TPM4, and MMP2 could be identified as novel drug targets in bone regeneration that may guide the development of new therapeutics.

### Future perspectives of postulated candidate proteins

In this meta-analysis, we thoroughly selected 51 key proteins based on stringent and comprehensible criteria. Additionally, we included further domain knowledge and studies not included in this meta-analysis to select the postulated multifunctional candidate proteins. The candidate proteins provide a thoroughly pre-selected list of biomolecules that will guide future experiment planning and further (pre-)clinical testing, as outlined below.

As such, biomarker candidates could be validated in controlled or even regulated (pre-)clinical cohorts that include healthy and bone-healing-impaired groups.^143^ It might be possible to retrospectively screen existing cohort studies not included in this meta-analysis. However, most existing studies focus on systemic bone impairments, for example, postmenopausal osteoporosis,^53^ that may not be applicable for non-diseased critical size-defects or fracture non-union related to other comorbidities like diabetes. For the suggested potential drug targets and their corresponding therapeutics the specific interactions need to be confirmed. For instance, Surface plasmon resonance spectroscopy or related methods allow the determination of protein/ligand-binding kinetics.^144^ These values can be used to estimate the dose of the postulated drugs and to evaluate whether this dose is below toxic concentrations based on known studies.^145–148^ Next, *in vitro* cellular bioassays may be applied to confirm whether a neutralizing effect on undesired outcomes can be triggered, and thus bone healing mechanisms may be supported. In the same manner, postulated bioactive bone healing supportive candidates need to be evaluated. However, the proteins suggested in this meta-study provide different levels of pre-existing experiments. For instance, IGFBP7 was previously proven to enhance fracture healing when administered intravenously^111^ and may be utilized for regulated preclinical studies directly. In contrast, ANXA1 is suggested to participate in EV-mediated healing mechanisms^70^ but direct evidence on bone regeneration is rare. Thus, extensive *in vitro* and *in vivo* studies need to be applied. This meta-analysis may also guide future decision making on suitable test strategies or analytical endpoints. For instance, ANXA1 was assigned as a tissue-specific CAP in *in vitro* cell-based and ECM studies as well as *in vivo* bone regeneration studies. In cell-based studies, ANXA1 forms an interaction network to nine other assigned tissue-specific CAPs including metabolically active proteins GAPDH, AKR1C3, and TGM2. Interestingly, six of the interconnected proteins are associated with programmed cell death. Both metabolic activity and apoptosis were previously shown to be tightly regulated in bone regeneration.^149,150^ In ECM studies ANXA1 is connected to three different tissue-specific CAPs with two of them being involved in biological adhesion (CD4 and S100A11). In regenerating bone ANXA1 forms a network with nine tissue-specific CAPs. Similar to cell-based studies, metabolically active proteins but related to glucose metabolism (ENO1, GOT1, and MDH2) were among the interconnected tissue-specific CAPs. Glucose metabolism was previously discussed as an important player to reprogram mature osteoblasts and contribute to osteoblast generation at bone injury sites.^151^ Additionally, eight of the non-ANXA1-connected tissue-specific CAPs in regenerating bone are components of exosomes. Exosomes are key drivers in bone regeneration by accumulating and transporting proteins and minerals to the newly formed ECM.^152^ Thus, ANXA1 is involved in a variety of biological processes, covering central metabolism, apoptosis, cell adhesion and exosome-mediated transport. However, not all functions can be covered in every tissue type. These findings make clear that (i) study types to test a novel designed biomaterial need to be selected depending on the research focus as well as aim and (ii) only the combination of different tissue types allows an overarching view on the vastness of molecular interactions. Further systems biological approaches are needed to fully understand the complex interplay and dynamics of different biological hierarchies and tissues in bone regeneration.

Upon thorough confirmation of the postulated bioactive factors and therapeutics, they can be tested in novel biomaterial innovations. As such, the postulated bioactive factors that are components of the bone ECM (e.g., CCN1, IGFBP7, LGALS1, and TGFBI) could be utilized as biomaterial coatings. Ideally, a bone biomaterial would only act as a temporary scaffold that will guide as well as enhance the intrinsic regenerative capacities and will be degraded upon healing.^8^ Different biodegradable materials were recently developed and calcium phosphate bioceramics are actually in clinical use.^153^ However, newly developed biodegradable synthetic polymers, such as poly-L-lactic acid (PLA), polycaprolactone (PCL), polylactic-co-glycolic acid (PLGA), poly (vinyl alcohol; PVA), and different composites with organic and inorganic materials provide promising results for future developments.^154–157^ Those synthetic biomaterials are available for additive manufacturing techniques allowing the production for patient-individualized scaffolds.^158^ Post-manufacturing processing of synthetic polymer-based biomaterials like wet chemical functionalizations, plasma surface modification, chemical crosslinking, or attachment of chemical linkers increase the hydrophilicity of the surface and create functional groups.^159–162^ Those functional groups can be utilized to covalently attach the postulated bioactive proteins onto newly developed bone biomaterials.

However, other suggested bioactive factors (e.g., ANXA1 and SERPINA1) that may act as soluble proteins and the postulated therapeutics should be considered to be released from the biomaterial into the regenerative niche. The time and spatially defined delivery of active substances via biomaterials was previously reviewed by Ferracini et al.^163^ Additionally, Montoya et al.^9^ visioned “smart” biomaterials with a responsive and autonomous release of its payload. The basis for this ambitious vision is provided by different delivery systems that were already developed. This includes direct absorption or imprinting of released factors in the biomaterial. However, this directly couples scaffold resorption and payload release which may not be intended for all bioactive proteins or drugs. Nanoparticles have become a versatile and adaptable delivery system. The choice and processing of biodegradable synthetic polymers allows the embedding of hydrophilic and hydrophobic proteins and drugs. Moreover, adaptable formulations allow a controlled, gradual and sustained release.^164^ Thus, already today different tools for releasing the suggested bioactive proteins or therapeutics are available for testing the postulated candidates *in vitro* and *in vivo*. Additionally, Zeng et al.^165^ reviewed the possibilities for a systemic but bone tissue-targeting delivery of therapeutics.

**Figure 7.**
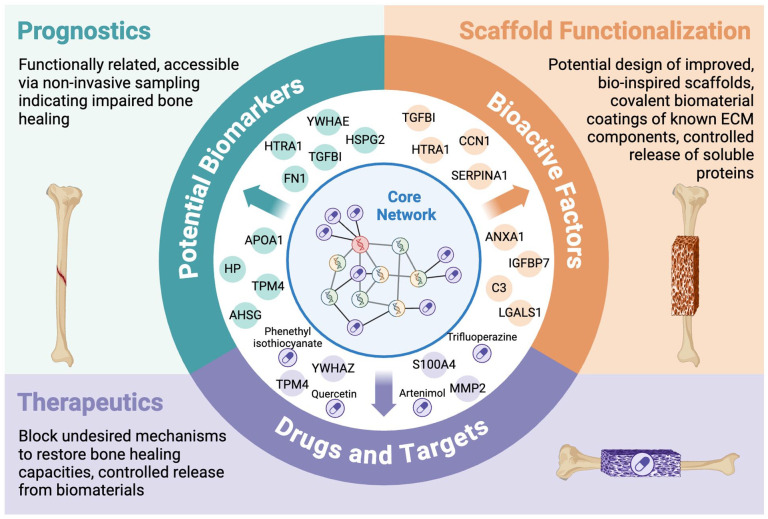
Schematic overview of novel multifunctional candidate proteins through meta-analysis of the collected proteomics data and their implications for (pre-)clinical research. All core network proteins were validated based on network topology and by including external domain knowledge to extract the suggested multifunctional candidate proteins. Only novel candidates not suggested by other research studies were included in this figure.

Further research will be necessary to prove the applicability of the here postulated multifunctional candidate proteins. Ultimately, this meta-analysis may guide biomarker discovery and bone biomaterial innovation (Figure 7).

### Limitations

We have implemented various measures to minimize the key limitations of meta-analyses, including publication bias, data availability, variability in study design and model selection, potential biases in biological databases, and inherent constraints in method validation. Studies with significant results are more likely to be published, leading to the potential underrepresentation of true negatives or less significant findings.^166^ This is a general problem that meta-studies encounter. We mitigated its impact by adhering to the PRISMA guidelines to capture a broader range of studies.^167^ However, we could only include studies with published raw data or providing complete statistics on protein quantification, by which we may have excluded potentially relevant, however less FAIR, studies. This limitation is inherent in the data availability landscape. Although the FAIR principle was recently introduced,^168^ older publications still tend to have missing data and were, thus, excluded from the meta-analysis. To encourage the inclusion of future datasets, we have made the scripts used in our analysis publicly available, enabling others to extend our analysis as new data becomes accessible (see *Implementation and Code Availability* statement). The included studies vary significantly in experimental design, tissue types, and quantification methods, for example, TMT, LFQ. While these differences could affect the comparability of the results,^169^ we leveraged this diversity by adopting a multi-tissue approach, which allows us to explore common biological processes across different tissue types and experimental designs. Additionally the studies vary in the format of the reported IDs. We addressed this by harmonizing protein identifiers^28^ and applying consistent re-analysis protocols (see Methods section: *Retrieval of Differentially Abundant Proteins and Meta-Study Harmonization*). In the same manner, mapping proteins across species, for example, human to rat, introduces potential inaccuracies, as orthologs may not fully represent functional equivalence between species.^170^ Additionally, some orthologs may be missing in studies with less frequently studied organisms. While this limitation is inherent in cross-species analysis and cannot be fully eliminated with current methods, we evaluated the accuracy of ID mapping and provided a detailed assessment of mapping quality (see Supplemental Figure 2). Ultimately, selected proteins were included into network and functional enrichment analyses. Both rely on pre-existing biological databases, for example, PPI networks, GO, KEGG, which can introduce bias toward well-studied proteins, leading to hub-node bias.^171^ The choice of algorithm, such as the Multi-Steiner Tree algorithm for network enrichment, approximates the minimum spanning tree, and varying the number of trees or tolerance levels could affect the resulting networks.^66,172^ While we cannot eliminate the bias inherent in biological databases, we addressed this by selecting well-curated databases and statistically validating the results with DIGEST to ensure meaningful networks.^29^

## Conclusion

To the best of our knowledge, this study is the first to combine proteomics analyses of diverse study types across multiple species to gain a novel viewpoint on protein-driven molecular processes in bone regeneration. We developed and successfully applied a framework to identify novel diagnostic biomarkers, bioactive candidates for biomaterial functionalizations and drug targets which could be leveraged to steer bone healing repurposing approved therapeutics. A vastness of more than 800 associated functions among commonly affected proteins in bone regeneration underscores the molecular complexity and dynamics that interplay in healing processes. This demands further research to be undertaken in the future, especially in disease models with altered physiology and endogenous healing capability.

Alongside our reported findings in the bone regeneration context, we provided a detailed tutorial that can serve as a blueprint for conducting meta-analyses across various proteomics research questions. Thus making it a versatile tool for researchers aiming to integrate and analyze complex proteomics data from multiple studies and species.

## Methods

The methodology underlying this study is presented in a comprehensive and structured manner, as outlined in the accompanying figure (Figure 2). From the initial data collection and meticulous preparation to the intricate network enrichment and subsequent validation, each phase is systematically detailed in the following.

### Data preparation

The foundation of robust data analysis lies in meticulous data preparation. This includes the intricate processes of study retrieval, careful selection, re-evaluation of raw data, and the harmonization of protein identifiers, ensuring a cohesive and accurate dataset for in-depth analysis.

#### Search strategy

For this study, we followed the Preferred Reporting Items for Systematic Reviews and Meta-Analyses (PRISMA) checklist^167^ which is uploaded as a Supplemental File (Supplemental Table 6). A dual strategy was applied consisting of surveys of publicly available proteomics raw data and previously published studies. Initially, the ProteomeXchange data repository^25^ was searched separately for each of the keywords “osteoblast,” “bone,” “plasma,” “serum,” “exosome,” and “extracellular vesicle” (last access 30th April 2021). All 1,012 cumulative hits (Supplemental Table 7) were considered for subsequent study selection. Additionally, a literature survey was performed on PubMed database (last accessed 17th December 2021) to extract pertinent publications applying proteomics analyses in the field of bone regeneration. The following search query was used, limiting the search results to the last 5 years: “proteomic”[Title/Abstract] AND (“osteoblast”[Title/Abstract] OR “bone”[Title/Abstract]). The query resulted in 526 publications (Supplemental Table 7) that were considered for further study selection.

#### Study selection

The identified 1012 and 526 studies were included in a selection process visualized in the PRISMA flowchart (Supplemental Figure 12) modified from the corresponding PRISMA2020 R package.^173^ Studies were screened for duplicates before title and abstract screening, which excluded the majority of the records (990 and 400, respectively). The remaining studies were assessed for full data access. Subsequently, a detailed full text analysis was applied.

#### Data re-evaluation for proteomics raw data

To make data directly comparable, studies with available raw data were re-analyzed using MaxQuant (version 1.6.3.3).^27^ Parameters for relative quantification were set as given in the original publications and included LFQ, SILAC, and TMT. All further parameters were fixed for all studies: experimental mass spectra were matched to reference proteomes of *Homo sapiens* or *Rattus norvegicus* both retrieved from UniProtKB. Trypsin/P was set as protease in specific mode allowing for a maximum of two missed cleavages. Carbamidomethylation of cysteine was set as fixed modification whereas oxidation of methionine and acetylation of protein N-terms were set as variable modifications. Mass tolerances were limited to 26 ppm for full scans in the first search as well as fragment ions and to 4.5 ppm for full scans in the main search. A minimum of two peptides including one unique peptide was necessary for protein inference. FDR for peptide-spectrum-matches, peptides and proteins were controlled to 0.05 by target/decoy evaluation. Parameter files and unprocessed protein output tables are available as Supplemental Information (Supplemental Data 1, Supplemental Table 8).

#### Retrieval of differentially abundant proteins and meta-study harmonization

For the retrieval of differentially abundant proteins (DAPs) diverging strategies were applied based on the source of studies. (i) DAPs of re-analyzed proteomics raw data were identified using Perseus (version 1.6.15.0).^174^ Therefore, data normalization and cutoff filtering (*p*-values, adjustment methods, log_2_-fold changes) were performed as given in the original publications. All study-specific filters, methods, and criteria are provided in the Supplemental Table 1. (ii) DAPs of studies not providing primary research data were directly extracted from published studies either from main text sections or available Supplemental Files. All DAPs were combined in a data table alongside further complementary information. Due to the diverse nature of the input lists (Supplemental Figure 1), the data table was processed via ProHarMeD^28^ prior to data analysis. During this process, proteins were filtered to only include IDs that are listed in the current version of UniProt^175^ (state: 03.11.2023) and were assigned to the correct organism using the organism-based filtering option. Subsequently, the filtered protein IDs were remapped to gene symbols, catering to studies that provided only gene symbols, thereby facilitating cross-study comparisons. For that, ProHarMeD utilized information from UniProt where the primary gene names were extracted. Given that gene symbols lack uniqueness and the primary gene symbol can vary depending on the database, it is crucial to standardize these symbols to ensure that the same gene is consistently referred to by the same name. For this, a third harmonization step, ProHarMeD’s reduction function was used, selecting Ensembl^176^ as the database source (Supplemental Table 1). Finally, to ensure consistency in the identification of genes across various species, it is essential to map the genes to a common reference species, here *Homo sapiens*. ProHarMeD utilizes the g:Profiler^30^ API to access information from the Ensembl database, facilitating the mapping of genes to their corresponding orthologs. ProHarMeD also features an automated logging function that tracks the effectiveness of each identifier conversion method used, aiding users in assessing the success rate of the conversion process. The results of these conversions, achieved through the four harmonization steps, are illustrated in Supplemental Figure 2.

### Selection of commonly affected proteins and network enrichment

Upon harmonization, the next step was to select CAPs that are consistently present across multiple studies. This step is designed to pinpoint proteins that are essential for network enrichment, thereby providing a deeper insight into biological functionalities. Identifying these CAPs allows for the construction of a cohesive network that highlights key pathways and interactions, enabling a more targeted approach for downstream analysis such as drug repurposing.

#### Selection of commonly affected proteins

To select relevant CAPs for network enrichment, different degrees of intersections were tested. Let protein p be a member of differentially abundant proteins (DAPs) and study s∈S , with S being the set of all studies. A function s(p) was defined to indicate the presence (one) or absence (zero) of protein p in study s .

Initially, all DAPs present in at least two studies were selected, expressed as $I\;:=\;p\;\in \;DAPs|\;\sum_{s\;\in S}\;s(p)\;\geq \;2$ , where I is the set of proteins p selected after the intersection analysis. However, this approach introduced a tissue-specific bias, given this analysis comprised studies from five different tissue types with varying numbers of studies. To address this, the selection criterion for a protein p was revised to include dependence on the set of tissue types t∈T , mandating that p must be present in at least half of the studies $s\;\in S_{t}$ , where $S_{t}$ is the set of studies from tissue type t , to be selected for I of tissue t defined as $I_{t}$ . This is defined as $I_{\forall t\in T}\;\;:\;=\;p\;\in \;DAPs\;|\;\sum_{s\;\in S_{t}}\;s(p)\;\geq \frac{\left|S_{t}\right|}{2}$ , which considers the varying number of studies across the different tissue types t∈T .

The process was further refined to account for varying study sizes by introducing a weight w for each protein p in study s , defined as $w_{p,s}\;=\frac{1}{\left|s\right|}$ . The total weight for a protein p across all studies $S_{t}$ , was then the sum of the weights, defined as $W_{p}=\sum_{s\;\in \;S_{t}}w_{p,s}$ . After intersecting proteins present in at least half of the studies of tissue t , a minimum weight threshold $W_{min}$ was established based on the lowest $W_{p}$ among the selected proteins $p\;\in \;I_{t}$ . Subsequently, every protein $p\;\notin \;I_{t}$ that satisfied $W_{p}\geq W_{min}$ , was also included in the final tissue-specific protein set $I_{t}$ , regardless of whether it was intersecting in at least half of the studies. This method is referred to as the Weighted Scoring Intersection (WSI) and will be used throughout the following sections (Supplemental Figure 3). The proteins of the tissue-specific protein sets $I_{t}$ will be referred to as tissue-specific CAPs throughout the study.

After selecting proteins for each tissue t using WSI, another subset of protein was extracted, defined as multi-tissue CAPs $I_{multi-tissue}$ , comprising those present in more than one final tissue-specific protein set $I_{t}\;\in I$ , defined as $I_{\mathrm{multi}-\mathrm{tissue}}:=\left\{p\;\in I\;:|\{\;t\in T|p\in I_{t}\}|>1\right\}$ .

#### Network enrichment of relevant CAPs

Network enrichment transforms the selected CAPs from isolated entities to a unified biological network. By incorporating established biological knowledge, this approach connects CAPs, potentially adding new connecting nodes for a more holistic view of protein interactions. This approach identifies key pathways and hubs, which are highly relevant for understanding complex biological processes. Hub nodes are central nodes, that is, crucial molecular players, with a potential over-arching impact on disease mechanisms. Hence, they represent *per se* ideal drug targets. Network enrichment, thus, prioritizes proteins crucial to network integrity, potentially revealing novel therapeutic targets that may not be identified through state-of-the art differential abundance analysis. For this, we employed NeDRex^23^ and Drugst.One,^24^ which are computational tools providing up-to-date methods for network exploration that have been systematically evaluated and benchmarked.^23,24,171^ Network enrichment was conducted on the pre-selected tissue-specific CAPs $\left\{I_{t}|t\in T\right\}$ and the multi-tissue CAPs $I_{multi-tissue}$ , using the Drugst.One^24^ Python package. This process aimed to construct a single connected component for each protein set, potentially revealing interacting mechanisms among the proteins. The NeDRex interaction graph $Graph_{nedrex}$ was employed for this purpose, derived from the NeDRexDB knowledgebase.^23^ NeDRexDB integrates a wealth of data from various biomedical databases, enhancing the robustness and comprehensiveness of the analysis. Among the databases integrated are OMIM,^177^ DisGeNET,^178^ UniProt,^175^ NCBI gene info,^179^ IID,^63^ MONDO,^83^ DrugBank,^180^ Reactome,^181^ and DrugCentral.^182^ This integration of multiple databases ensures a wide-ranging and in-depth coverage of gene-gene interactions, enabling the coverage and connection of all CAPs compared to networks from single databases such as STRING, Biogrid, and IID (Supplemental Figures 5 and 6). To identify the necessary interaction partners (proteins) that link each set of CAPs $i\;\in I_{t\;\in T}$ and $i\;\in I_{multi-tissue}$ respectively within the network together into a single connected component, the Multi-Steiner Tree algorithm^66^ was utilized. Given the graph ${\mathrm{Graph}}_{\mathrm{nedrex}}=(\mathrm{Nodes},\;\mathrm{Edges})$ , this algorithm identifies a subset of protein $\mathrm{Node}s^{\prime }$ that connects all proteins in i where the sum of the weights of the $\mathrm{Edge}s^{\prime }$ is minimized in the new subgraph $G\mathrm{rap}h^{\prime }=(\mathrm{Node}s^{\prime },\;\mathrm{Edge}s^{\prime })$ , with $i\subseteq \mathrm{Node}s^{\prime }$ . The parameters used for the algorithm included a tolerance of 5 and a hub penalty of 0.5, which controls how much the resulting trees may expand the number of edges and how central nodes are penalized. Additionally, five Steiner trees were computed to provide variations in the minimum spanning subnetwork, balancing approximation quality and runtime. The PPI dataset used for the network construction was sourced from NeDRexDB, providing the most comprehensive knowledge-base as outlined above (Supplemental Figure 5). The Multi-Steiner Tree algorithm thus resulted in single-tissue networks and a core network that minimizes the total weight of the edges while connecting all proteins of interest.

### Validation of network enrichments and ranking of nodes

The validation of network enrichments involves integrating biological annotations with graph attributes analysis, including centrality measures. Additionally, the relevance of proteins in bone tissue and their biological accessibility are evaluated. This validation stage is further extended by the identification of potential drug targets, underscoring opportunities for repurposing existing therapeutic options. These steps ensure that the networks represent cohesive biological systems with practical implications for bone health and treatment. Note, this validation process is applied exclusively to the core network, as it encompasses proteins that are consistently identified across multiple tissue types, providing a comprehensive and biologically relevant framework for identifying potential biomarkers, bioactive factors, key therapeutic targets and potential drug repurposing opportunities.

#### Analysis of internal functional coherence

The reference-free protein subnetwork validation feature of DIGEST^29^ was utilized to analyze the internal functional coherence of the candidate mechanism by using GO and KEGG annotations. Each protein’s contribution is measured through a functional score, calculated as the mean Jaccard index across gene pairs. A higher functional score indicates better functional coherence. We used permutation testing with 1000 iterations to evaluate the significance of network enrichments. The functional score is assessed via an empirical *p*-value, comparing the observed score against recalculated functional scores generated from a background model that randomizes the input while preserving the protein count and connectivity of the original subnetwork. Additionally, each protein’s impact on network coherence is evaluated individually; a positive value indicates a beneficial effect, while a negative value suggests the opposite. Each protein’s contribution score is determined as the median of the GO and KEGG annotation scores. This thorough analysis helps identify proteins that significantly contribute to the functional integrity of the network.

#### Statistical enrichment and graph attributes

The second validation step utilized g:GOSt from g:Profiler^30^ for functional enrichment analysis. This analysis identifies the overrepresentation of functional categories like biological processes, cellular components, molecular functions, or pathways in a specified protein list. The method compares annotations against a background set and calculates overlaps for each category. The statistical significance of these overlaps is assessed using tests like the hypergeometric test, represented by *p*-values. Functional categories were considered significantly enriched if the *p*-value was less than 0.05 after the Benjamini-Hochberg correction for multiple testing. The outcome is a list of significantly enriched functional categories and the contributing genes. Additionally, an enrichment score for each protein p is calculated based on their involvement in these categories. The resulting four enrichment scores are used to rank each gene, where a higher score indicates more significant enrichment. Finally, the median of those five ranks is calculated to yield a single ranking value for each protein.

In addition to the previously explained scorings, each protein p was evaluated based on the graph attributes of the network graph G . These graph attributes provide insights into the roles of proteins in the network topology, and are calculated using the igraph library.^31^ The attributes included closeness centrality, measuring the proximity of a gene to all others in the network; betweenness centrality, indicating how often a protein appears on the shortest path between two other proteins; degree, representing the number of direct connections a protein has; harmonic centrality, summing the reciprocals of the shortest path distances from a protein to all others; and PageRank, which assigns importance based on the number and quality of links to the protein. Similar to the enrichment scores, the graph attribute scores were utilized to rank proteins, with higher scores indicating better performance.

Note, that the rankings were then summarized into one combined enrichment and network ranking, by calculating the median of the respective ranks.

#### Accessibility and bone abundance ranking

Additionally, proteins were scored regarding their potential accessibility by liquid-biopsy utilizing the HPA^32^ and Vesiclepedia database.^33^ Proteins were ranked based on their secretion status (“secreted to blood”) and blood concentration evaluated by MS (both information retrieved from HPA). Further, proteins were ranked by the number of observing studies (general) and the number of observational blood plasma or serum studies. The bone abundance score was implemented to estimate the relevance of proteins in bone tissue utilizing information from HPA. Proteins were ranked based on their secretion status (“secreted to extracellular matrix”), normalized TPM (transcripts per million) counts in bone marrow as well as normalized TPM counts in the U-2 OS cell line.

Just as with the enrichment and network rankings, the accessibility and bone abundance rankings were also summarized into combined ranks.

#### Drug repurposing

Drug repurposing offers a fast and cost-effective alternative to traditional drug discovery, significantly reducing the time and resources needed to bring treatments to clinical use. It allows for a direct connection between molecular insights and existing drug candidates, accelerating the identification of effective treatments. This approach is especially valuable in targeting complex diseases, where multiple pathways can be addressed simultaneously.^183–185^

The implementation of Drugst.One^24^ played a pivotal role in the process of the previously described network enrichment, providing up-to-date and validated methods for network exploration.^23,24,171^ Initially, this tool was utilized to enrich the network with new candidate genes, ensuring the formation of a connected subnetwork. This connected subnetwork is valuable because it provides a unified framework of interacting proteins, revealing key interaction partners that might not be apparent from individual datasets.

Our network-based drug repurposing approach builds on this framework, leveraging the extensive biological knowledge embedded in PPI networks. By analyzing these networks, we target critical nodes and pathways that are central to disease mechanisms, rather than isolated proteins. This enhances the likelihood of identifying drugs that have a broader, more effective impact on the biological system, making it a robust strategy for drug discovery.

Following that, Drugst.One was employed to investigate the proteins within the single-tissue networks for their potential as drug targets. In addition to identifying potential drug targets, Drugst.One was also applied to identify the top 20 drugs targeting the proteins within the network. This scoring of the drugs is based on the trust scores assigned by TrustRank, a version of the PageRank algorithm adapted by Drugst.One, which highlights the drugs most likely to influence the proteins of interest in the network. TrustRank operates by crawling the network based on user-selected seed nodes and ranks visited nodes, with a damping factor ranging from 0 to 1 controlling how far the crawler explores the network. The damping factor is set by default to 0.85, where the larger the factor, the larger the proportion of the network that is considered.

The methodology was extended to the core network, with one major difference: rather than restricting the number of drugs to 20 based on score, this approach included all possible drugs, allowing a greater scope for the study.

## Supplemental Material

sj-docx-1-tej-10.1177_20417314241295332 – Supplemental material for Meta-analysis of proteomics data from osteoblasts, bone, and blood: Insights into druggable targets, active factors, and potential biomarkers for bone biomaterial design Supplemental material, sj-docx-1-tej-10.1177_20417314241295332 for Meta-analysis of proteomics data from osteoblasts, bone, and blood: Insights into druggable targets, active factors, and potential biomarkers for bone biomaterial design by Johannes R Schmidt, Klaudia Adamowicz, Lis Arend, Jörg Lehmann, Markus List, Patrina SP Poh, Jan Baumbach, Stefan Kalkhof and Tanja Laske in Journal of Tissue Engineering

sj-docx-2-tej-10.1177_20417314241295332 – Supplemental material for Meta-analysis of proteomics data from osteoblasts, bone, and blood: Insights into druggable targets, active factors, and potential biomarkers for bone biomaterial design Supplemental material, sj-docx-2-tej-10.1177_20417314241295332 for Meta-analysis of proteomics data from osteoblasts, bone, and blood: Insights into druggable targets, active factors, and potential biomarkers for bone biomaterial design by Johannes R Schmidt, Klaudia Adamowicz, Lis Arend, Jörg Lehmann, Markus List, Patrina SP Poh, Jan Baumbach, Stefan Kalkhof and Tanja Laske in Journal of Tissue Engineering

sj-txt-9-tej-10.1177_20417314241295332 – Supplemental material for Meta-analysis of proteomics data from osteoblasts, bone, and blood: Insights into druggable targets, active factors, and potential biomarkers for bone biomaterial design Supplemental material, sj-txt-9-tej-10.1177_20417314241295332 for Meta-analysis of proteomics data from osteoblasts, bone, and blood: Insights into druggable targets, active factors, and potential biomarkers for bone biomaterial design by Johannes R Schmidt, Klaudia Adamowicz, Lis Arend, Jörg Lehmann, Markus List, Patrina SP Poh, Jan Baumbach, Stefan Kalkhof and Tanja Laske in Journal of Tissue Engineering

sj-xlsx-3-tej-10.1177_20417314241295332 – Supplemental material for Meta-analysis of proteomics data from osteoblasts, bone, and blood: Insights into druggable targets, active factors, and potential biomarkers for bone biomaterial design Supplemental material, sj-xlsx-3-tej-10.1177_20417314241295332 for Meta-analysis of proteomics data from osteoblasts, bone, and blood: Insights into druggable targets, active factors, and potential biomarkers for bone biomaterial design by Johannes R Schmidt, Klaudia Adamowicz, Lis Arend, Jörg Lehmann, Markus List, Patrina SP Poh, Jan Baumbach, Stefan Kalkhof and Tanja Laske in Journal of Tissue Engineering

sj-xlsx-4-tej-10.1177_20417314241295332 – Supplemental material for Meta-analysis of proteomics data from osteoblasts, bone, and blood: Insights into druggable targets, active factors, and potential biomarkers for bone biomaterial design Supplemental material, sj-xlsx-4-tej-10.1177_20417314241295332 for Meta-analysis of proteomics data from osteoblasts, bone, and blood: Insights into druggable targets, active factors, and potential biomarkers for bone biomaterial design by Johannes R Schmidt, Klaudia Adamowicz, Lis Arend, Jörg Lehmann, Markus List, Patrina SP Poh, Jan Baumbach, Stefan Kalkhof and Tanja Laske in Journal of Tissue Engineering

sj-xlsx-5-tej-10.1177_20417314241295332 – Supplemental material for Meta-analysis of proteomics data from osteoblasts, bone, and blood: Insights into druggable targets, active factors, and potential biomarkers for bone biomaterial design Supplemental material, sj-xlsx-5-tej-10.1177_20417314241295332 for Meta-analysis of proteomics data from osteoblasts, bone, and blood: Insights into druggable targets, active factors, and potential biomarkers for bone biomaterial design by Johannes R Schmidt, Klaudia Adamowicz, Lis Arend, Jörg Lehmann, Markus List, Patrina SP Poh, Jan Baumbach, Stefan Kalkhof and Tanja Laske in Journal of Tissue Engineering

sj-xlsx-6-tej-10.1177_20417314241295332 – Supplemental material for Meta-analysis of proteomics data from osteoblasts, bone, and blood: Insights into druggable targets, active factors, and potential biomarkers for bone biomaterial design Supplemental material, sj-xlsx-6-tej-10.1177_20417314241295332 for Meta-analysis of proteomics data from osteoblasts, bone, and blood: Insights into druggable targets, active factors, and potential biomarkers for bone biomaterial design by Johannes R Schmidt, Klaudia Adamowicz, Lis Arend, Jörg Lehmann, Markus List, Patrina SP Poh, Jan Baumbach, Stefan Kalkhof and Tanja Laske in Journal of Tissue Engineering

sj-xlsx-7-tej-10.1177_20417314241295332 – Supplemental material for Meta-analysis of proteomics data from osteoblasts, bone, and blood: Insights into druggable targets, active factors, and potential biomarkers for bone biomaterial design Supplemental material, sj-xlsx-7-tej-10.1177_20417314241295332 for Meta-analysis of proteomics data from osteoblasts, bone, and blood: Insights into druggable targets, active factors, and potential biomarkers for bone biomaterial design by Johannes R Schmidt, Klaudia Adamowicz, Lis Arend, Jörg Lehmann, Markus List, Patrina SP Poh, Jan Baumbach, Stefan Kalkhof and Tanja Laske in Journal of Tissue Engineering

sj-xlsx-8-tej-10.1177_20417314241295332 – Supplemental material for Meta-analysis of proteomics data from osteoblasts, bone, and blood: Insights into druggable targets, active factors, and potential biomarkers for bone biomaterial design Supplemental material, sj-xlsx-8-tej-10.1177_20417314241295332 for Meta-analysis of proteomics data from osteoblasts, bone, and blood: Insights into druggable targets, active factors, and potential biomarkers for bone biomaterial design by Johannes R Schmidt, Klaudia Adamowicz, Lis Arend, Jörg Lehmann, Markus List, Patrina SP Poh, Jan Baumbach, Stefan Kalkhof and Tanja Laske in Journal of Tissue Engineering
