# Supplementary material for: Meta-analysis of proteomics data from osteoblasts, bone, and blood: Insights into druggable targets, active factors, and potential biomarkers for bone biomaterial design
Source: J Tissue Eng. 2024 Nov 29;15:20417314241295332. doi: 10.1177/20417314241295332 (PMC11605762; doi:10.1177/20417314241295332)
Supplement: sj-docx-1-tej-10.1177_20417314241295332 – Supplemental material for Meta-analysis of proteomics data from osteoblasts, bone, and blood: Insights into druggable targets, active factors, and potential biomarkers for bone biomaterial design [file sj-docx-1-tej-10.1177_20417314241295332.docx]

# **Supplementary Figures**


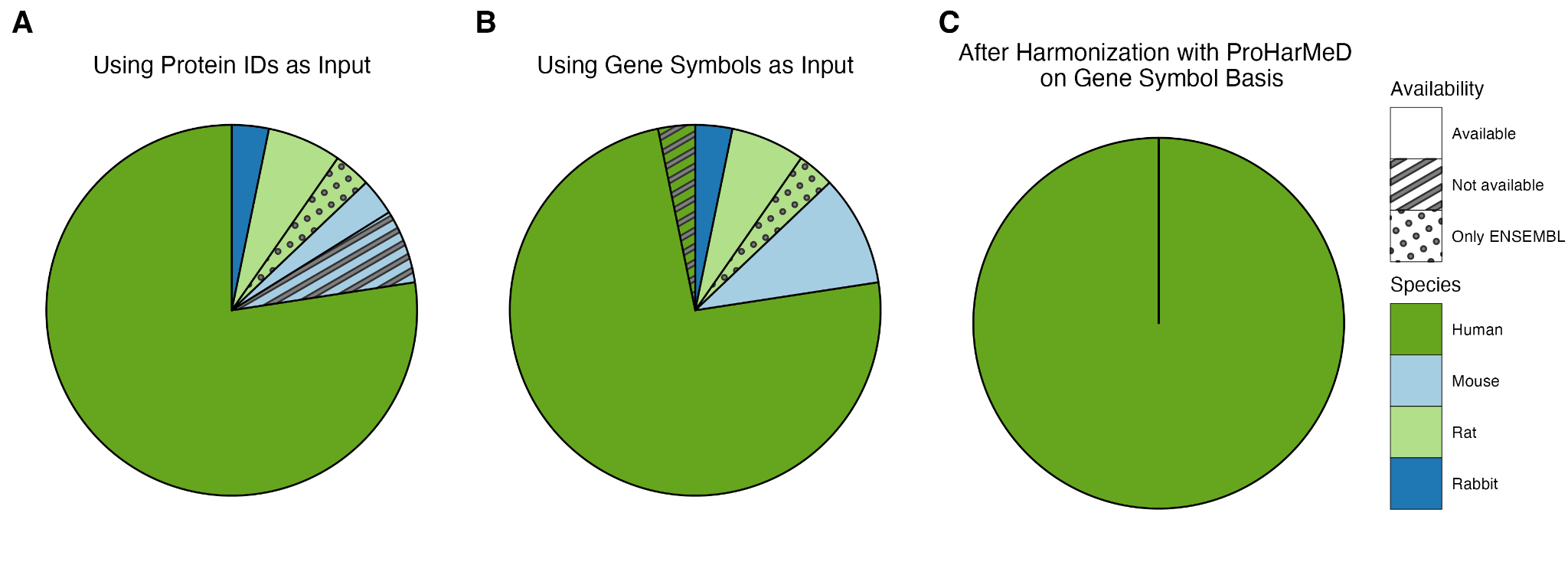


**Supplementary Figure 1: Harmonization impact on selected multi-species studies.** Heterogeneity of the studies on protein ID (A) and gene symbol levels (B), respectively. Using the supplied protein IDs or gene symbols of the selected studies originating from multiple species, the different input lists are highly heterogeneous. (C) Uniform mapping of inputs to human gene symbols by the application of the harmonization tool ProHarMeD.


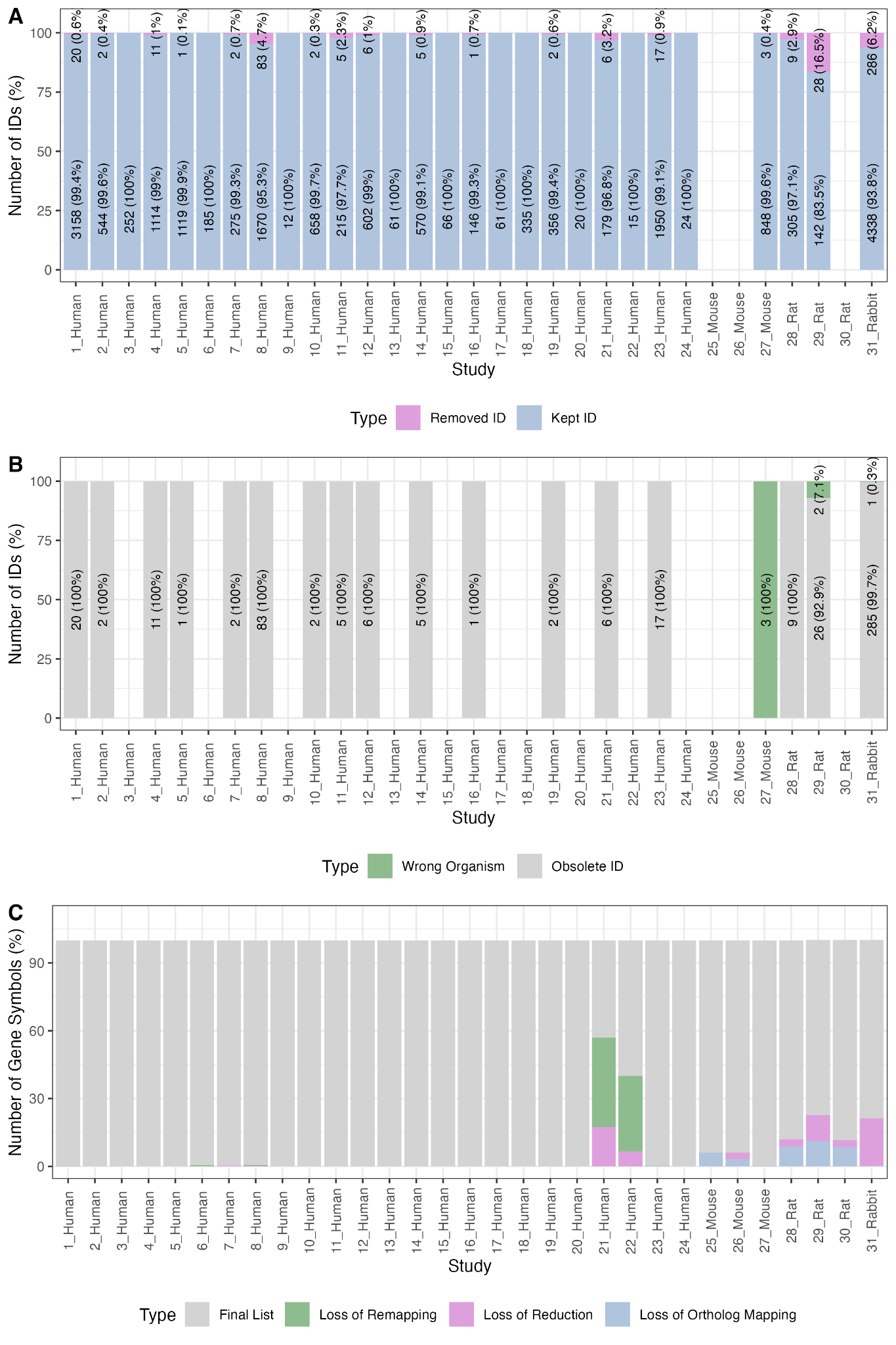


**Supplementary Figure 2: Graphical summary of conversion results of ProHarMeD.** The DAPs of the different studies were processed via ProHarMeD. Proteins were filtered to only include IDs that are listed in the current version of UniProt (state: 03.11.2023). The percentages of removed protein IDs during this filtering step are shown in (A) with the reason of removal displayed in (B). The filtered protein IDs were then remapped to gene symbols, standardized to consistent gene symbol naming, and non-human gene symbols were mapped to their corresponding orthologs. The percentages of removed gene symbols throughout these three steps are shown in (C).


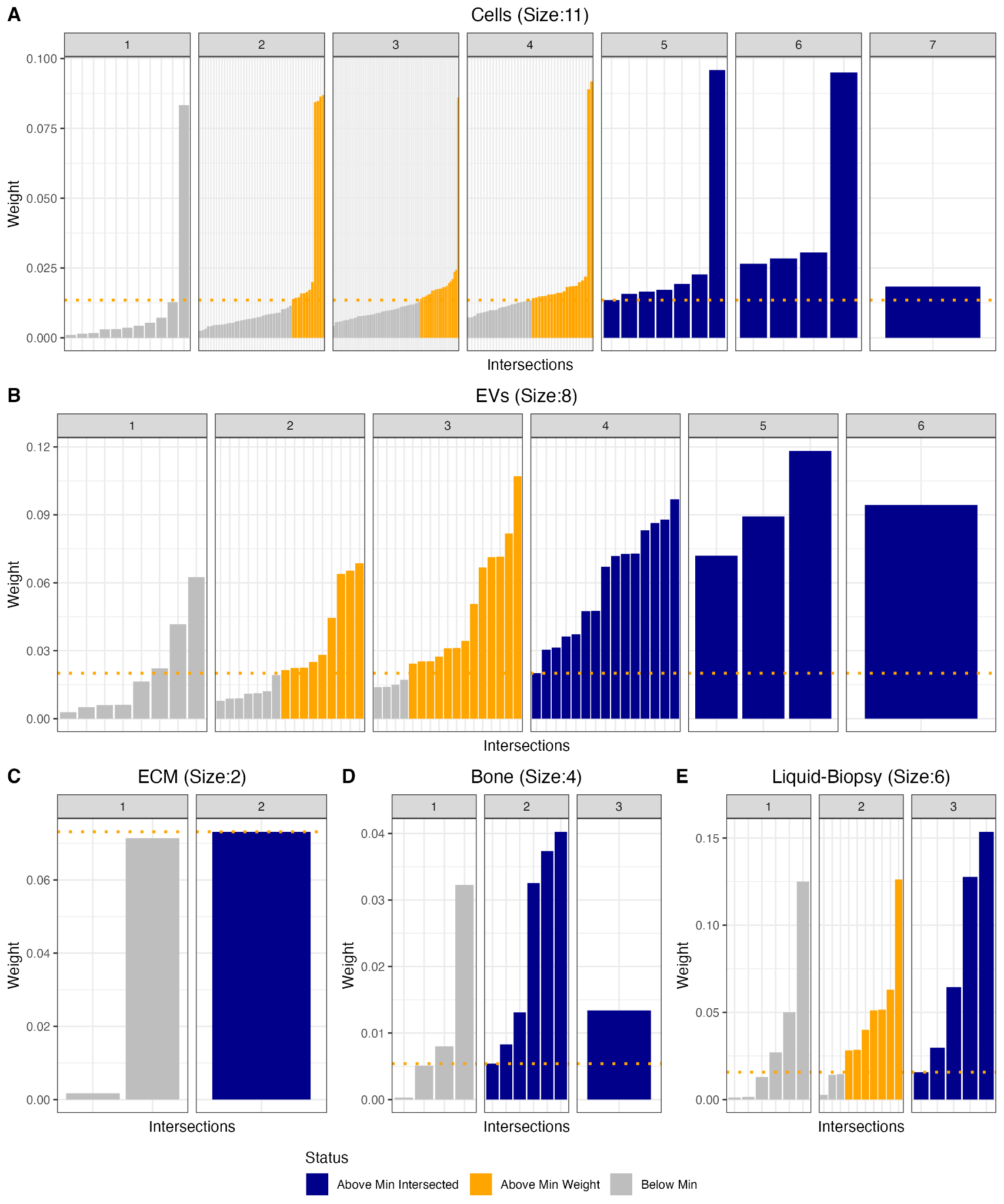


**Supplementary Figure 3: Weighted intersection filtering.** All cumulative proteins were filtered based on weighted intersections per tissue type. Proteins with re-identification rates > 50 % in studies per tissue type (blue) were included for further analysis. The minimum weight (bar height) of those protein sets was calculated and set as a threshold. All protein sets exceeding this threshold and with re-identification in at least two studies were further included (yellow). Weighted intersections were separately determined for (A) cells, (B) extracellular vesicles (EVs), (C) extracellular matrix (ECM), (D) bone, and (E) liquid biopsy.

**
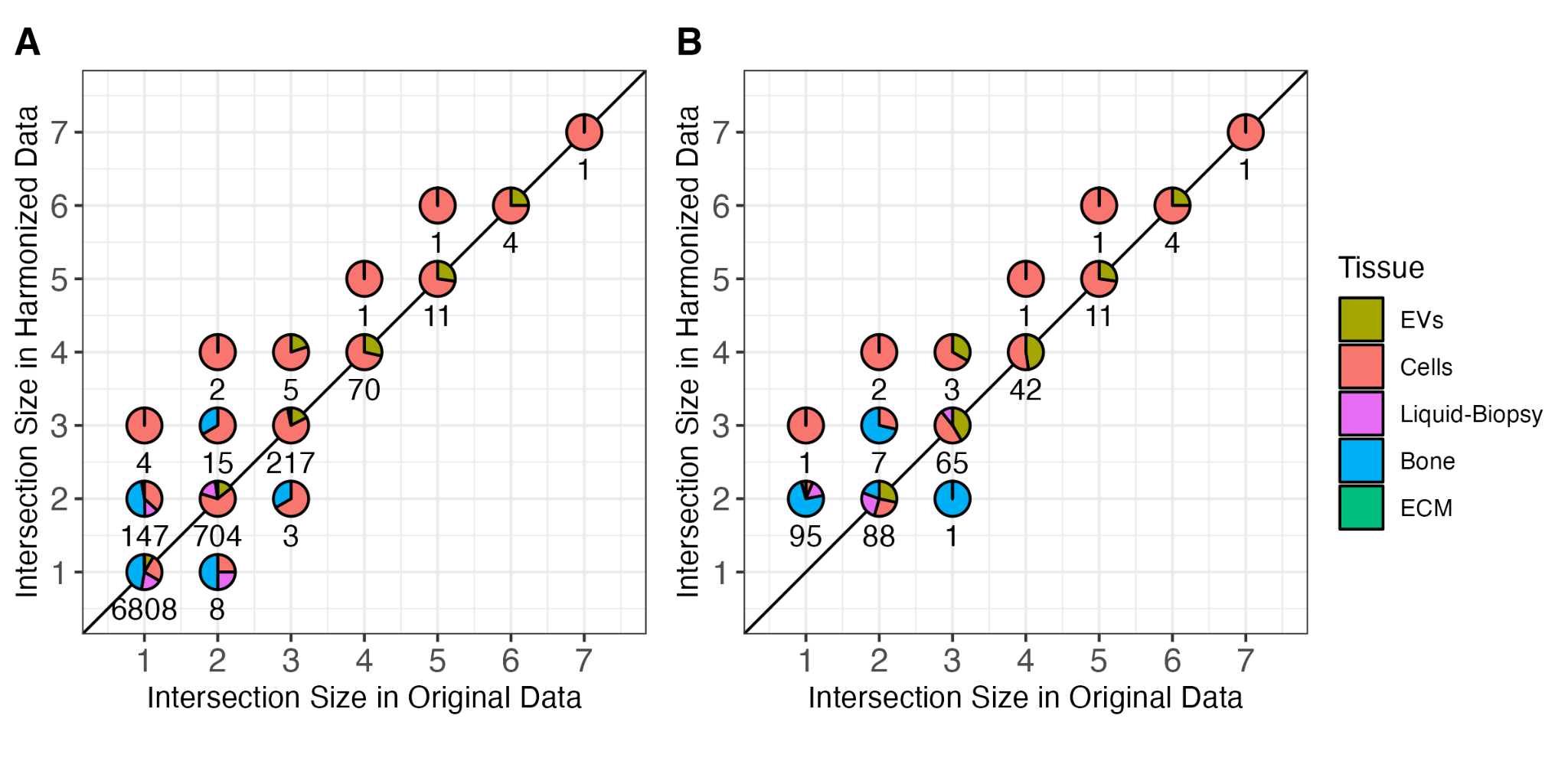
**

**Supplementary Figure 4: Comparison of intersection sizes pre- and post-harmonization.** In (A), the impact of harmonization across all data intersections is assessed, while (B) focuses on intersections resulting after the application of the Weighted Scoring Intersection (WSI) method. Each point aggregated into a pie chart corresponds to a gene symbol identified in both the original and harmonized data. The x-axis quantifies the study occurrence frequency of each gene symbol in the original data, whereas the y-axis represents this metric in the harmonized data. Points aligning along the diagonal represent gene symbols whose occurrence rates remain unchanged through the harmonization process.

**
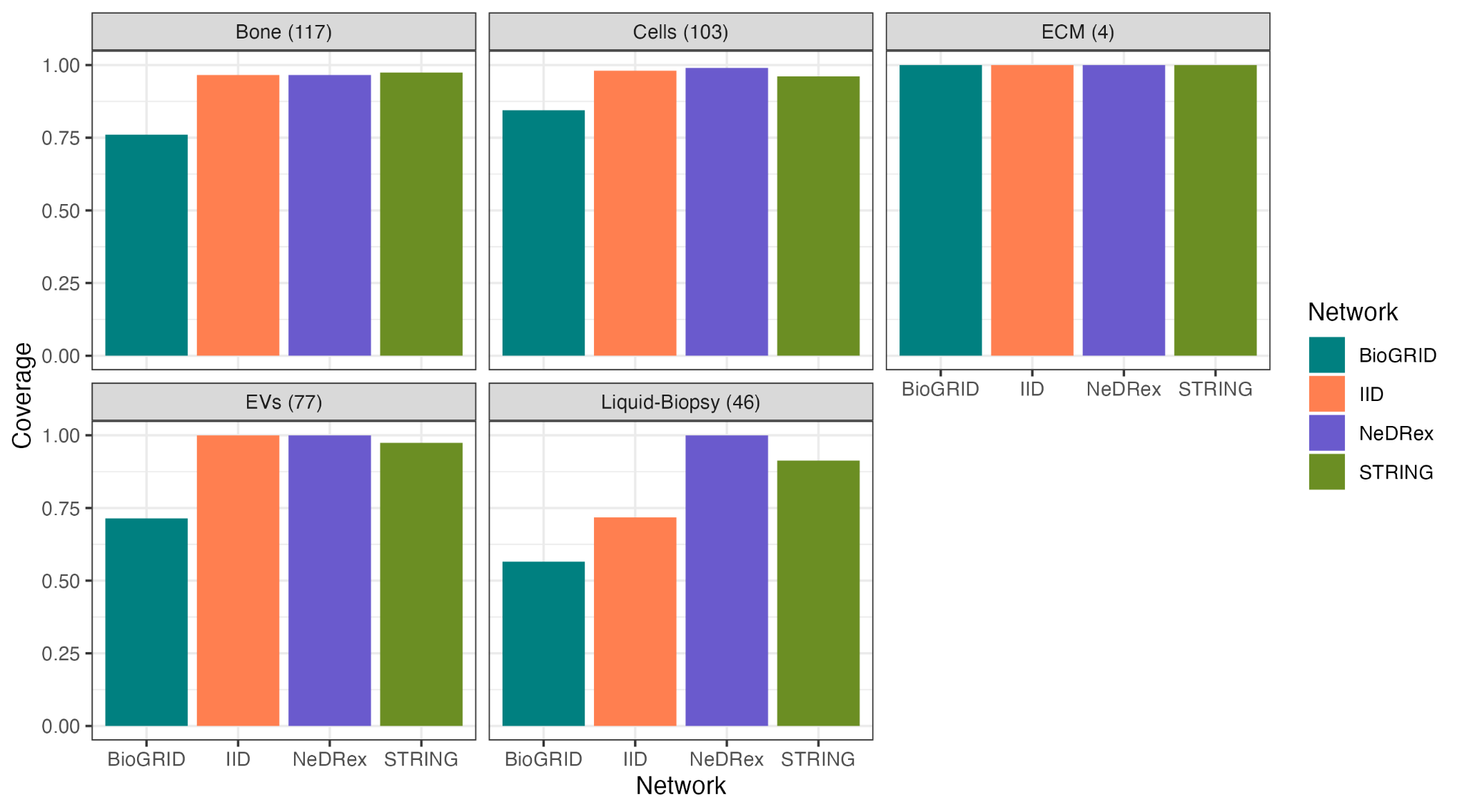
Supplementary Figure 5: Comparative coverage analysis of BioGRID, IID, NeDRex, and STRING networks across the different cell/tissue types.** Comparing the gene coverage of each network in encapsulating the desired differential abundant genes (DAPs) pertinent to each cell/tissue type (number indicated in parentheses) reveals that NeDReX ensures the coverage and connection of all DAPs) compared to networks from single databases such as STRING, BioGRID, and IID.

**
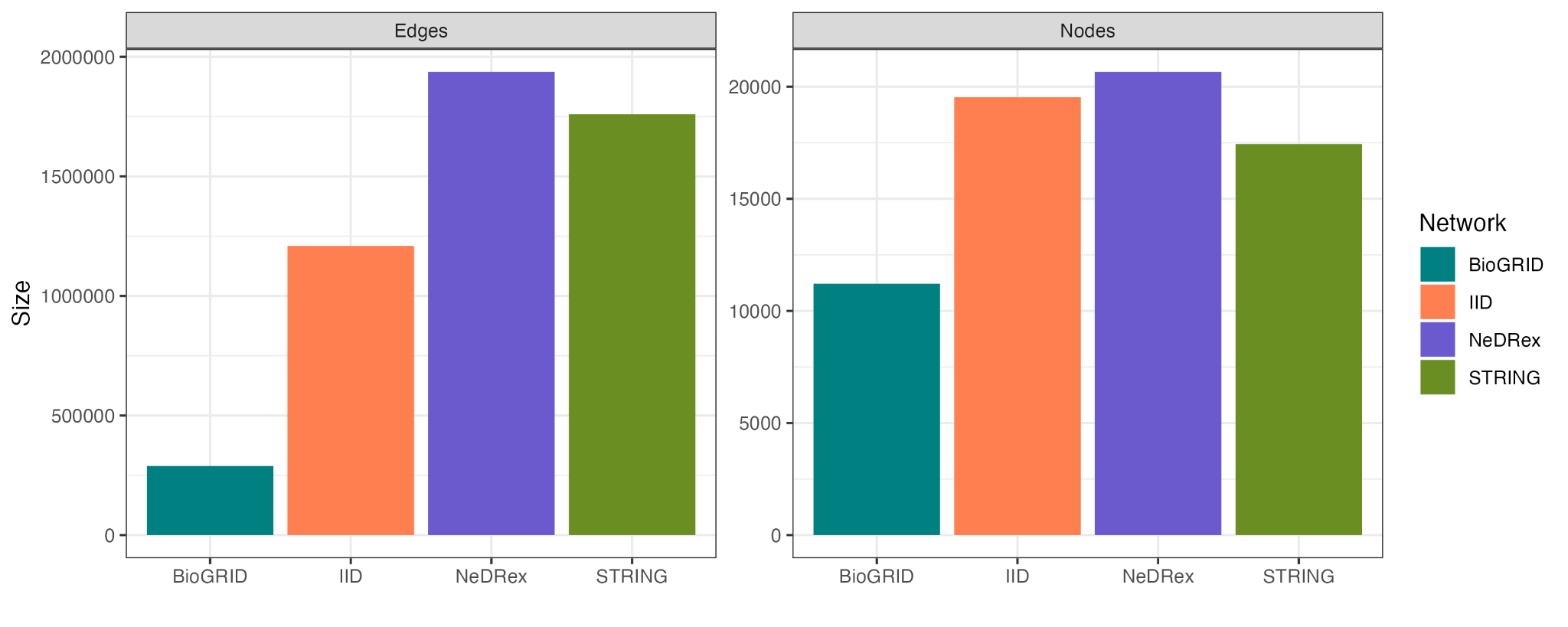
Supplementary Figure 6: Quantitative network comparison.** Comparing the network sizes of BioGRID, IID, NeDRex, and STRING, the NeDReX network is highlighted for having the greatest number of both nodes and edges.

**
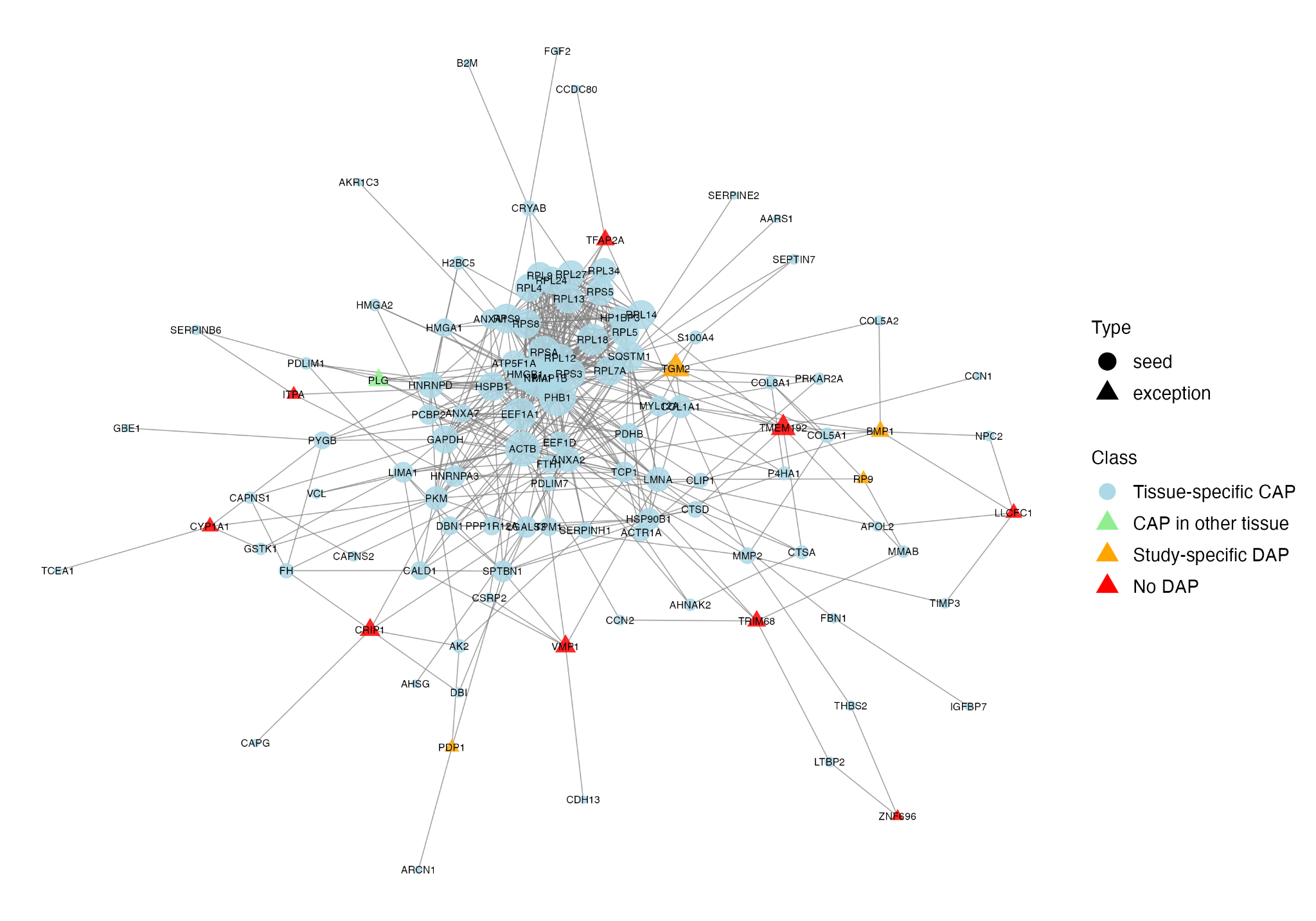
Supplementary Figure 7: Network of cellular origin.** The commonly affected proteins (CAPs) of cellular origin (tissue-specific CAPs) were used as seeds in network enrichment to construct a single connected network. The added proteins, i.e. exception nodes, necessary to connect the seeds are further separated into three categories: proteins that meet the criteria for CAPs in another tissue after WSI filtering (CAP in other tissue), proteins that were listed as a differential abundant protein (DAP) in a study but eliminated during WSI filtering (study-specific DAP), and novel proteins that have not been reported as DAP in any of the selected studies (no DAP). Legend applies to all Supplement Figures 7-11.

**
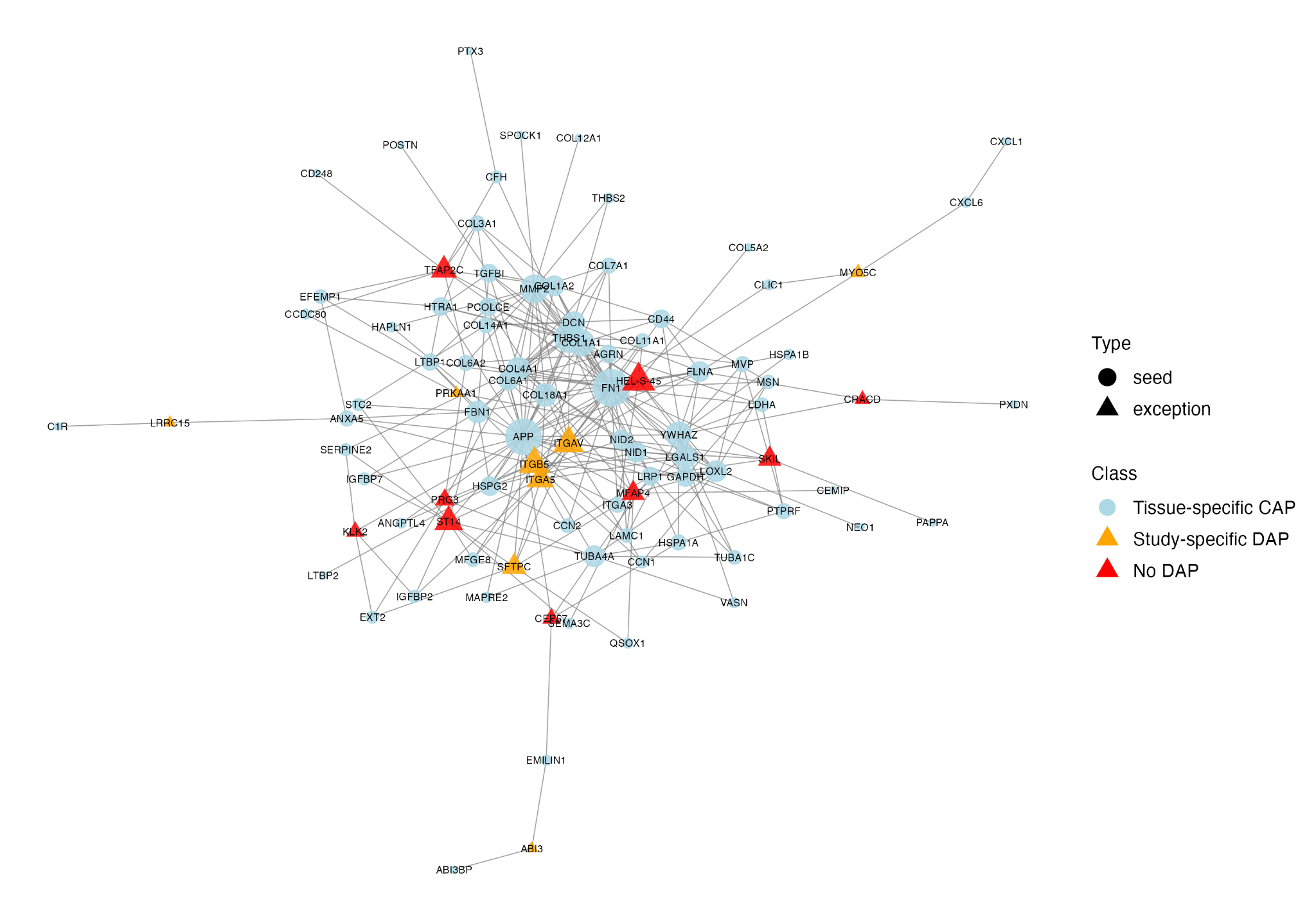
Supplementary Figure 8: Network of EV-focused *in vitro* analysis.**

**
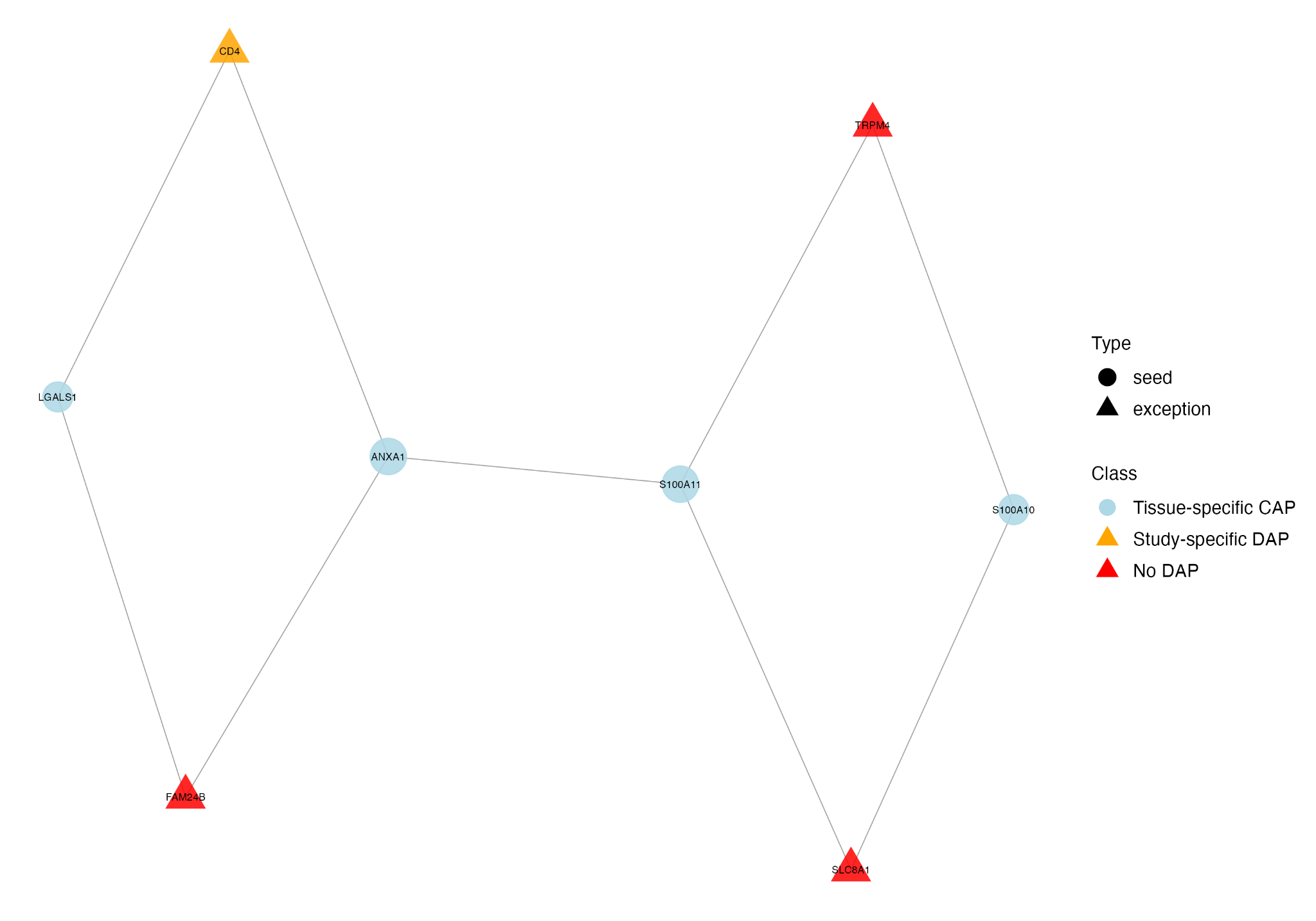
Supplementary Figure 9: Network of ECM-focused analysis.**

**
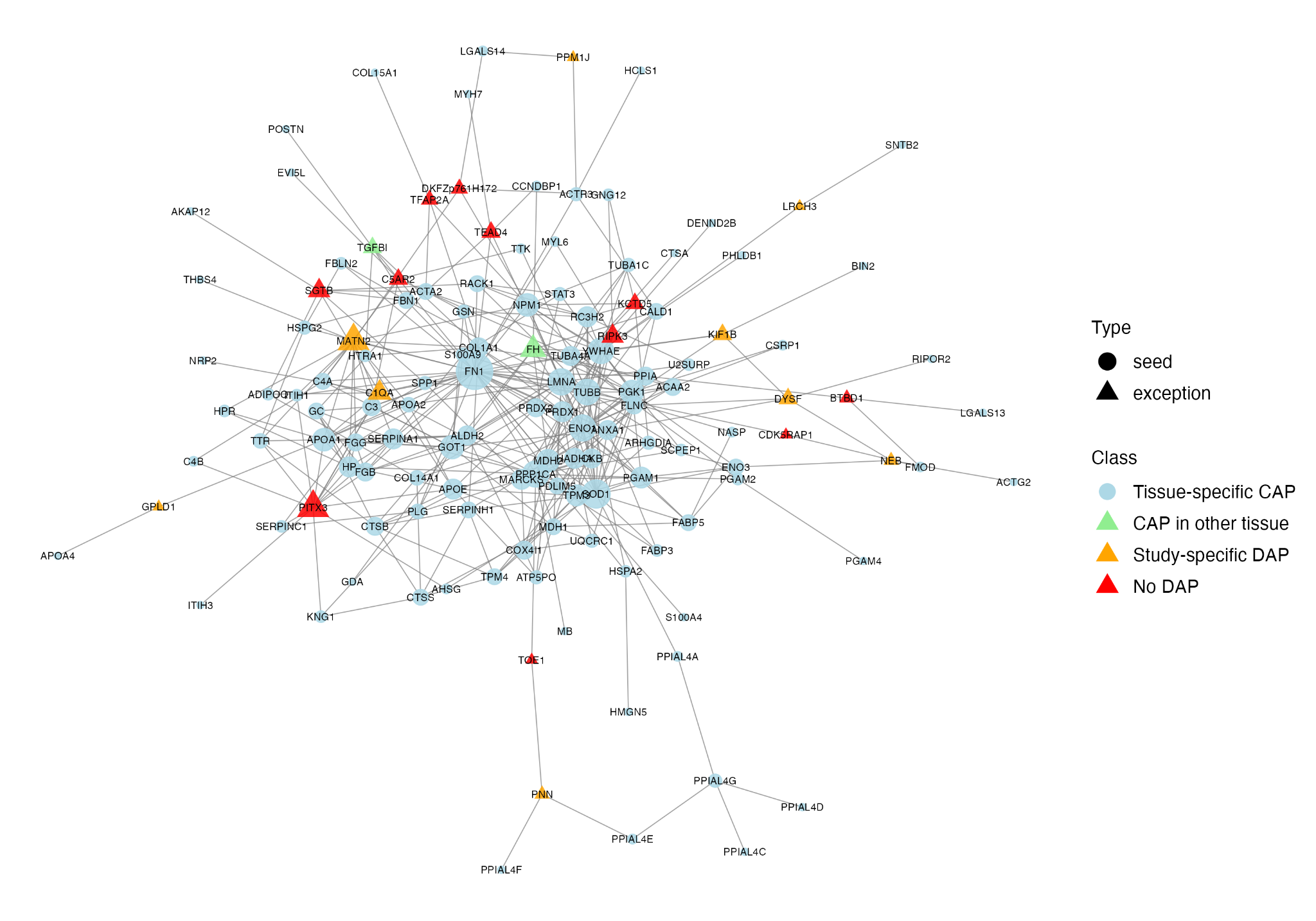
Supplementary Figure 10: Network of *in vivo* analysis of bone healing.**

**
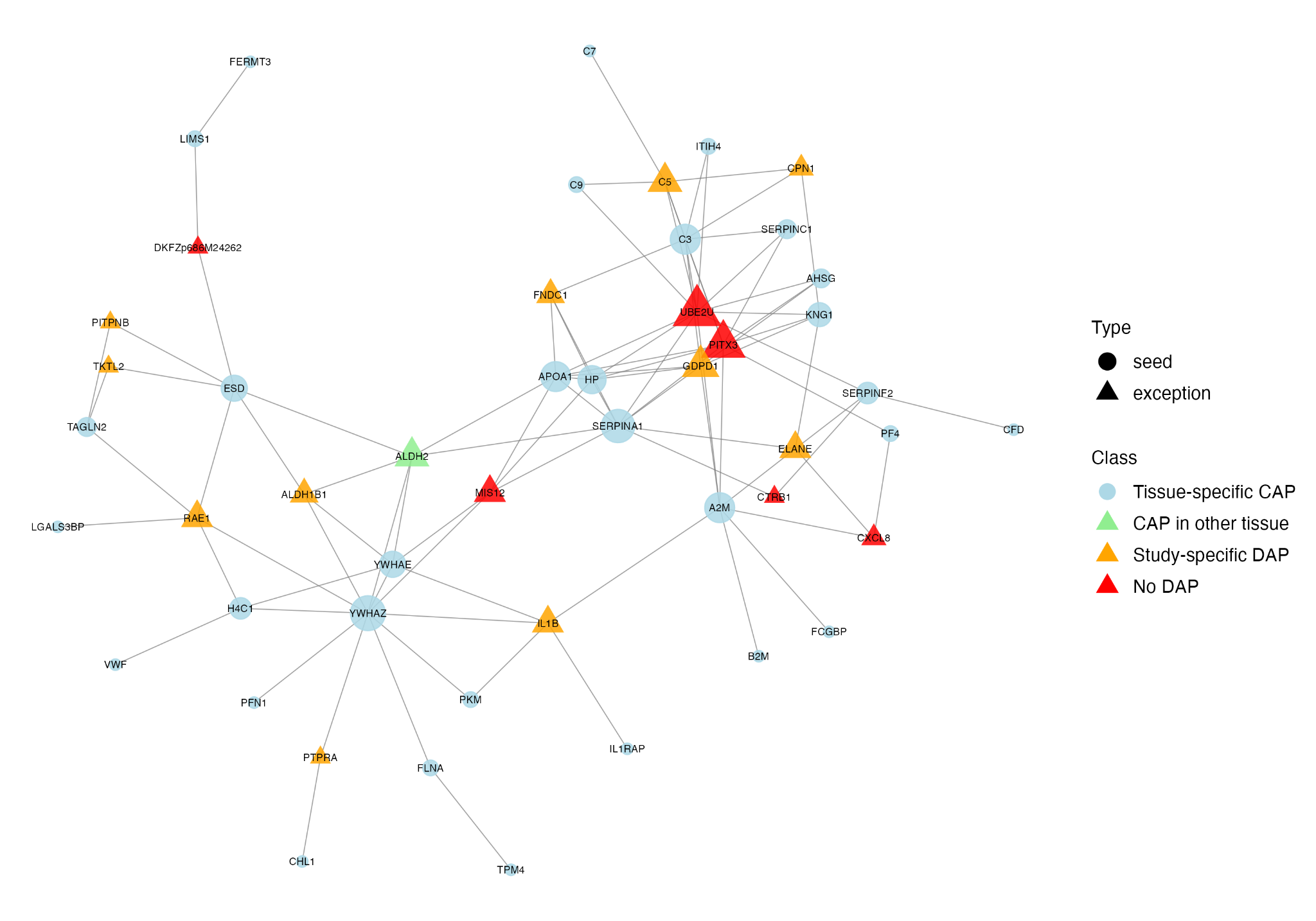
Supplementary Figure 11: Network of liquid biopsy studies.**


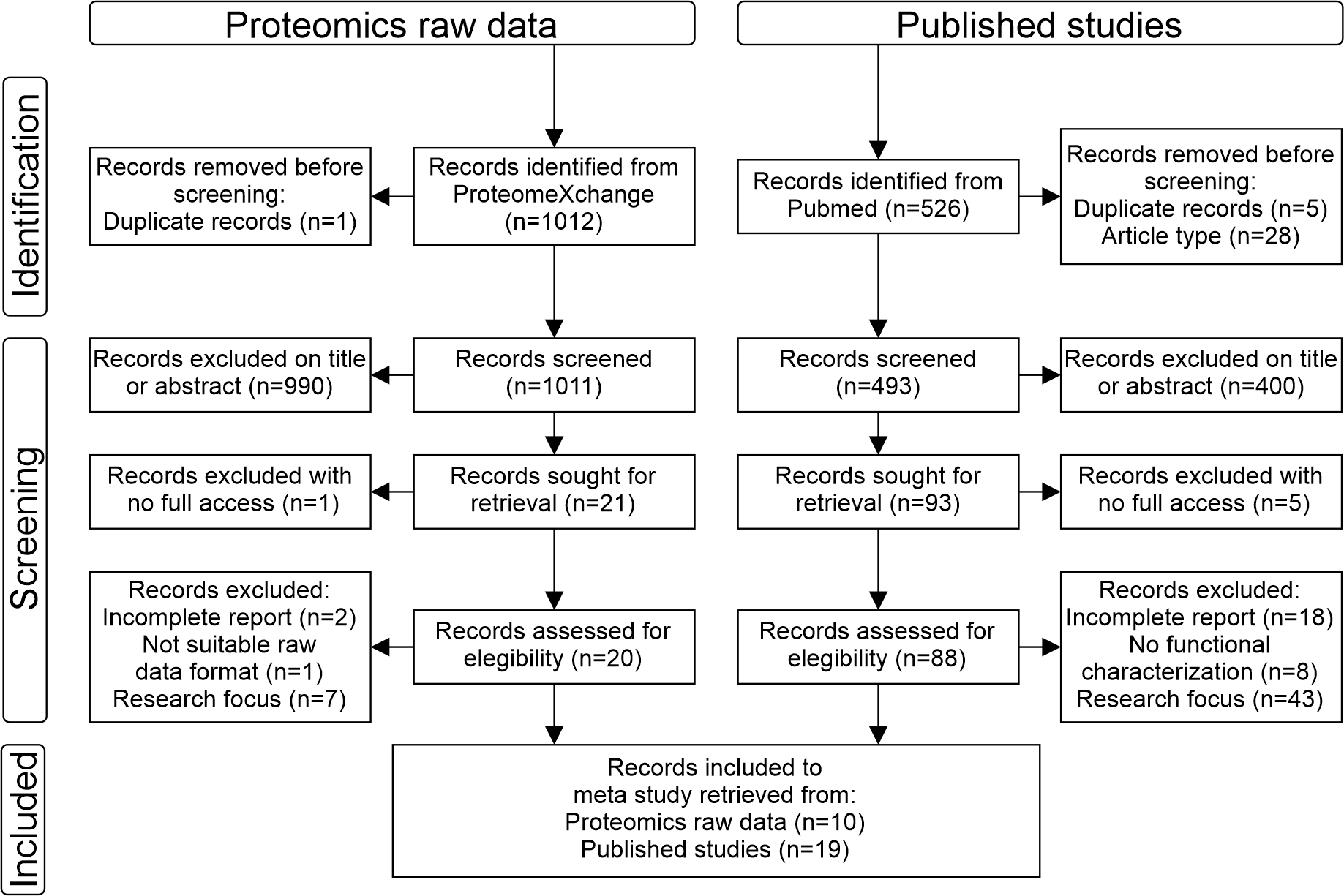


**Supplementary Figure 12.** **Study selection.** Overview of exclusion and inclusion flow for identified studies. Duplicate entries and published studies other than original research articles were excluded. The remaining records were screened based on their titles and abstracts. Only studies that involved a proteomics analysis of either osteoblasts, bone tissue or collected blood plasma/serum (including extracellular vesicles) after perturbations, healing-supporting interventions, or longitudinal observations of the cell differentiation or healing progress were further included for full data access check. The remaining records were assessed for eligibility in detail. Studies providing incomplete reporting, i.e., impossible assignment of raw data to study entities based on the given documentation or insufficient reporting of affected proteins. Raw data could also be excluded for not matching the requirements needed for integrated MaxQuant analysis. PubMed-retrieved studies were only kept if a functional characterization of perturbations was included. If all requirements were fulfilled, full text analysis of the publications was applied to confirm the matching research question to the above-mentioned criteria. The remaining ten and 19 studies were included in the here described meta-analysis. The data were extracted by two independent reviewers. (**Supplementary Table 1**).
